# Pan-Plastome Evolution and Metabolite Variation Provide Insights to Conservation of the Tibetan Medicinal Plant *Mirabilis himalaica*

**DOI:** 10.3390/plants15111691

**Published:** 2026-05-30

**Authors:** Yuxuan He, Nan Lin, Beier Duan, Jinhao Wang, Xiankun Wang, Zeyuan Cao, Song Song

**Affiliations:** 1 College of Life Science, Henan Agricultural University, Zhengzhou 450046, China; yuxuanhhee@outlook.com (Y.H.); 17396327178@163.com (B.D.); wjh3295957935@163.com (J.W.); 19836155621@163.com (Z.C.); 2 Henan Engineering Research Center for Osmanthus Germplasm Innovation and Resource Utilization, Henan Agricultural University, Zhengzhou 450046, China; 3 State Key Laboratory of Plant Diversity and Specialty Crops, Kunming Institute of Botany, Chinese Academy of Sciences, Kunming 650201, China; 4 College of Landscape Architecture, Henan Agricultural University, Zhengzhou 450046, China; wangxiankunx@163.com

**Keywords:** conservation units, genetic diversity, metabolites pattern, *Mirabilis himalaica*, pan-plastome

## Abstract

*Mirabilis himalaica* is an endemic Tibetan medicinal plant distributed from the Western Himalaya to the Hengduan Mountains, highly regarded for its abundant flavonoids. Traditional knowledge holds that its medicinal properties vary considerably with geographic origin, yet the genetic and metabolic basis of this differentiation remains poorly understood. Here, we integrated plastome resequencing of 134 individuals from 23 populations with metabolomic and transcriptomic analyses of three representative sites to investigate population genetic variation and flavonoid metabolic differentiation. Pan-plastome revealed a typical quadripartite structure (154,232–154,422 bp) containing 113 unique genes across *M. himalaica*. A total of 620 SNVs, 171 indels, and four small inversions were identified from the pan-plastome, and further analyses based on these variants supported the delineation of four genetic lineages across all individuals. Overall genetic diversity was high (*H*_T_ = 0.985, *H*_S_ = 0.580), with majority variation occurring among groups (71.038%). Both IBD and IBE analyses found a significantly positive correlation between genetic distance and geographic and environmental distance (IBD: r = 0.348, *p* = 0.001; IBE: r = 0.219, *p* = 0.016). Flavonoids represented the most abundant metabolites (19.5%) and showed significantly higher accumulation in high-altitude populations, where key biosynthetic genes (e.g., *CHS*) were upregulated. Notably, these altitude-associated metabolic patterns were observed independently of the plastome-based genetic lineages. Together, we propose defining four evolutionary lineages as conservation units and prioritizing populations with unique haplotypes. This study provides critical genomic resources for provenance tracing, quality evaluation, and conservation management of this endangered Tibetan medicinal plant, and offers preliminary insights into the parallel patterns of pan-plastome variation and altitude-related metabolic differentiation, though without evidencing a direct causal link between them.

## 1. Introduction

*Mirabilis himalaica* (Edgew.) Heimerl (Nyctaginaceae) is an endemic herbal medicine ranging from the Western Himalayas to the Hengduan Mountains [1], which mainly grows at the mountains edges, shrub grasslands, and rock crevices of dry-warm river valleys [2]. The genus *Mirabilis* L. comprises approximately 60 species, predominantly distributed in temperate and tropical regions of North and South America, with only a single species occurring in Asia—namely, *M. himalaica* [1,2,3]. According to classical Tibetan medical literature, *M. himalaica* is widely used in traditional Tibetan folk medicine and is recognized as one of the well-known Tibetan medicinal plants among the “Five Roots” [4]. Accordingly, it is ascribed therapeutic functions in warming the kidney, promoting tissue regeneration, inducing diuresis, and eliminating “yellow water” [5,6]. Modern chromatographic analyses have identified that its bioactive constituents mainly include flavonoids, steroids, and phenylpropanoid derivatives, exhibiting diverse potential pharmacological activities in anti-inflammatory, antitumor, and antioxidant effects [6,7,8]. Notably, rotenoids have been recognized as one of the key bioactive medicinal compounds in *M. himalaica*, which is included in isoflavonoids with prominent anticancer properties [9,10]. In particular, Linghu et al. showed that rotenoid induces S-phase cell cycle arrest in A549 lung cancer cells, suggesting that its anticancer activity may involve the inhibition of cell proliferation [6]. Despite its considerable medicinal value, *M. himalaica* has been listed as a first-class rare and endangered Tibetan medicinal plant with Near Threatened status in China due to overharvesting, habitat destruction, and overgrazing [11].

In traditional medicinal applications and market circulation, the properties of *M. himalaica* are thought to differ considerably with geographic origin [12]. For example, crude materials from Tibet are generally regarded as superior to those from Sichuan and Yunnan [12]. Nevertheless, these empirical perceptions have caused the adulteration and false labeling of geographical origin in the market [13]. Therefore, establishing a rapid and robust identification system is crucial for the quality evaluation, geographical tracing, and conservation of wild *M. himalaica* resources. Morphological examinations of the roots have revealed differences between wild and cultivated materials in surface color, texture, and odor, as well as internal anatomical structures [12]. Previous studies using nuclear ribosomal ITS sequencing indicated genetic differentiation between wild and cultivated *M. himalaica* from different geographical origins and habitat conditions, but its low-resolution largely limited precise identification [14]. In addition, HPLC-based analysis reveals that wild *M. himalaica* contains higher levels of key bioactive constituents than cultivated samples by chromatographic characteristics [12]. Despite traditional use focusing on the roots, metabolomic evidence indicates that leaves contain greater amounts of pharmacologically active metabolites, implying that aboveground organs are capable of revealing metabolic differences associated with geographical origin [7]. However, the patterns of altitudinal or geographic variation in *M. himalaica* leaf-derived bioactive compounds have yet to be elucidated.

Pan-plastomes integrate plastome data from multiple individuals, enabling the identification of both highly conserved core regions and variable hotspots [15,16]. Compared to the limited molecular markers used in traditional DNA barcodes, pan-plastomes with abundant nucleotide variations can improve the accuracy of species identification in medicinal plants [17,18]. This approach has been effective for taxonomy and phylogenetic studies of medicinal plants, even among closely related medicinal plants, such as *Polygonatum* Mill., *Euchresta* Benn., *Atractylodes* DC., and *Gentiana* (Tourn.) L. L. [19,20,21,22]. However, these plastome studies have typically included only a few representative samples and focused primarily on interspecific differences, thus providing limited insights into intraspecific genetic variation and the underlying evolutionary processes [23,24]. Recent studies have revealed that pan-plastomes contribute to population-level genetic variation associated with geographical origin, supporting provenance tracing and conservation of medicinal plant resources [25,26]. For instance, pan-plastome analyses of *Forsythia suspensa* detected abundant intraspecific genetic variation in single-nucleotide variants (SNVs) and structural variants, enabling us to identify genetic clusters [27]. Similarly, pan-plastomes of the medicinal plant *Dioscorea nipponica* have identified variation hotspots and resolved intraspecific phylogenetic relationships, highlighting substantial genetic variation at the population level [28]. Nevertheless, few studies on medicinal plants have integrated pan-plastome variation with geographically genetic differentiation, particularly regarding the potential association between pan-plastome variation and bioactive compounds. In this study, we conducted pan-plastome sequencing on 134 individuals from 23 *M. himalaica* populations with the following objectives: (1) to assess intraspecific genetic variation and genetic structure across populations; (2) to identify candidate genetic markers based on pan-plastomes that capture geographic differentiation to evaluate their utility for provenance tracing and conservation management; and (3) to integrate transcriptome and metabolite analyses across elevation gradients to evaluate whether chemical compositions exhibit geographic variation. This research will provide genomic resources for future studies of this medicinally valuable species and reveal intraspecific genetic variation patterns relevant to conservation and breeding.

## 2. Results

### 2.1. Comparative Analysis of the Pan-Plastome in M. himalaica

**Figure 1:**
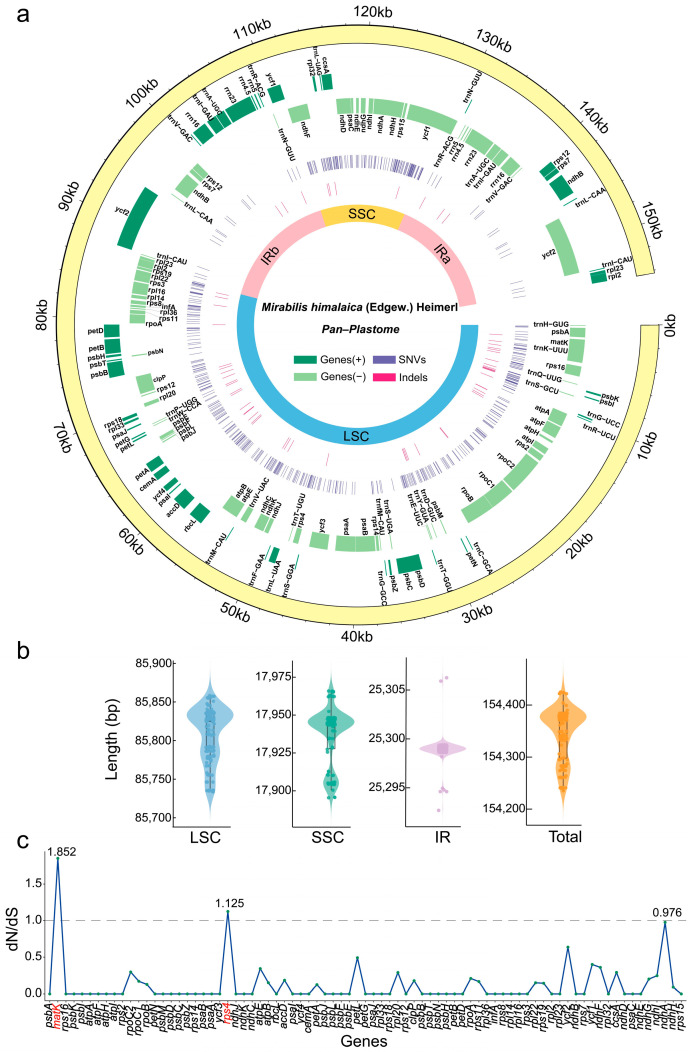
The pan-plastome evolution of *Mirabilis himalaica.* (**a**) Genomic structure and mutation distribution in the *M. himalaica* pan-plastome. (**b**) Violin plots showing the length variation in the large single-copy (LSC) region, the small single-copy (SSC) region, the inverted repeat (IR) regions, and the total pan-plastomes among 134 individuals; each dot represents one individual. (**c**) Ratio of non-synonymous substitution rate (dN)/synonymous substitution rate (dS) in the pan-plastome.

The pan-plastomes of all 134 *M. himalaica* individuals exhibited a typical quadripartite structure, consisting of a large single-copy (LSC) region, a small single-copy (SSC) region, and a pair of inverted repeats (IRa and IRb) (Figure 1a and Figure S1). The total plastome size ranged from 154,232 to 154,422 bp, with an average of 154,343 bp. The LSC region ranged from 85,733 to 85,858 bp (mean 85,808 bp); whereas, the SSC region spanned from 17,896 to 17,966 bp (mean 17,937 bp), and the IR region ranged from 25,293 to 25,306 bp (mean 25,299 bp) (Figure 1b and Table S1). The overall GC content was 36.0%, with the IR regions showing the highest GC content (42.70%). A total of 113 genes were annotated across the *M. himalaica* plastomes, including 79 protein-coding genes, 30 tRNA genes, and four rRNA genes (Table S2). The *ndhD* gene was identified as a pseudogene in three populations from the Nepal lineage, where a single-nucleotide mutation causes premature termination of the coding sequence. Pan-plastome comparison revealed that *ycf1* and *rpl2* were located at the IRa-SSC (JSA) and IRb-LSC (JSB) boundaries, respectively. Selection pressure analysis revealed that *matK* and *rps4* presented dN/dS ratios exceeding one; whereas, all remaining genes exhibited dN/dS values below one (Figure 1c).

### 2.2. Single-Nucleotide Variations and Small Structural Variations

**Figure 2:**
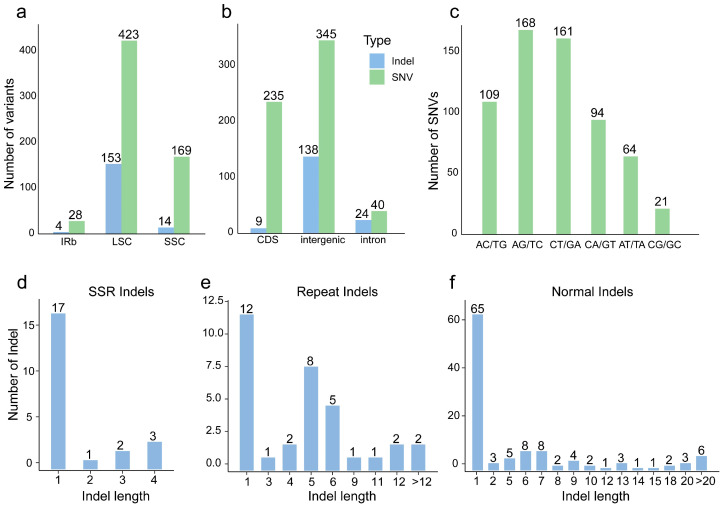
Nucleotide variations in the *Mirabilis himalaica* pan-plastome. (**a**) Genome-wide distribution of single-nucleotide variants (SNVs) and (**b**) indels. (**c**) Composition of SNVs types. (**d**–**f**) Length frequency distributions of three indel categories (SSRs, repeat indels, and normal indels).

A total of 620 SNVs were identified, including 617 biallelic sites and three triallelic sites. Among these, 152 were singleton sites and 468 were parsimony-informative sites (Table S3). Transitions (Ts) comprised most of SNVs (329 sites), with the most common substitution types being from T to C and from A to G (Figure 2c). The distribution of SNVs across the *M. himalaica* pan-plastome was highly heterogeneous of quadripartite structure. The LSC region contained the largest number of SNVs (423 SNVs, 68.22%), followed by the SSC region (169 SNVs, 27.26%) and IR regions (28 SNVs, 4.52%; Figure 2a). Meanwhile, intergenic spacer regions harbored the majority of SNVs (345), followed by coding regions (235) and introns (40). Across 57 protein-coding genes with SNVs, *ycf1* retained the highest number of SNVs (61 SNVs), followed by *rpoC2* (13 SNVs) and *ccsA* (13 SNVs; Figure 2b and Table S4).

Among 171 indels identified in *M. himalaica* pan-plastome, 23 were microsatellite-related indels, 34 repeat-related indels, and 114 normal indels (Figure 2d–f). Most indels were located in intergenic spacer regions, with only nine occurring in coding regions (two each in *accD, petL*, and *ccsA*, one each in *matk*, *ycf1* and *rpl22*). The three indel types differed significantly in length. Normal indels were predominantly 1 bp, though six exceeded 20 bp. Microsatellite-related indels were mostly 1 bp (17/23, 73.91%), arising from poly A/T repeats. The *trnS-GCU–trnG-UCC* region exhibited the highest number of microsatellite-related indels, with five identified. Only one microsatellite-related insertion (1 bp in the *rpoC1* intron) was detected, and none occurred in coding regions. Repeat-related indels ranged from 1 bp to 37 bp, with 1 bp being the most common (12/34, 35.29%). The longest repeat-related indels were in the *atpA-atpF* intergenic region, and two were in coding regions (*ycf1* and *rpl22)*. Four small inversions were identified, with all of these located in intergenic regions. Inverted regions were 4–18 bp, whereas the flanking stems were 17–34 bp (Table S5).

The numbers of potentially informative characters (PICs) for each genomic region are summarized in Table S4. A total of 58 protein-coding genes and 60 intergenic regions contained PICs, with values ranging from 1 to 62. The percentage of PICs relative to the length of each region ranged from 0.046% to 10%. Among the protein-coding genes, PIC ratios varied from 0.046% in *ycf2* to 4.522% in *ycf1*, with an average value of 0.562%. In contrast, intergenic regions exhibited markedly higher levels of variation, with PIC ratios ranging from 0.125% in the *ycf2-ndhB* spacer to 10% in the *psbT-psbN* spacer, with an average of 1.591%. Several intergenic regions exhibited particularly high PIC densities and may represent mutation hotspots, including *psbT-psbN*, *ccsA-ndhD*, and *ycf1*, which showed PIC ratios exceeding 4%. These highly variable regions may serve as potential molecular markers for phylogenetic reconstruction and population genetic studies.

### 2.3. Population Structure and Haplotype Distribution

**Figure 3:**
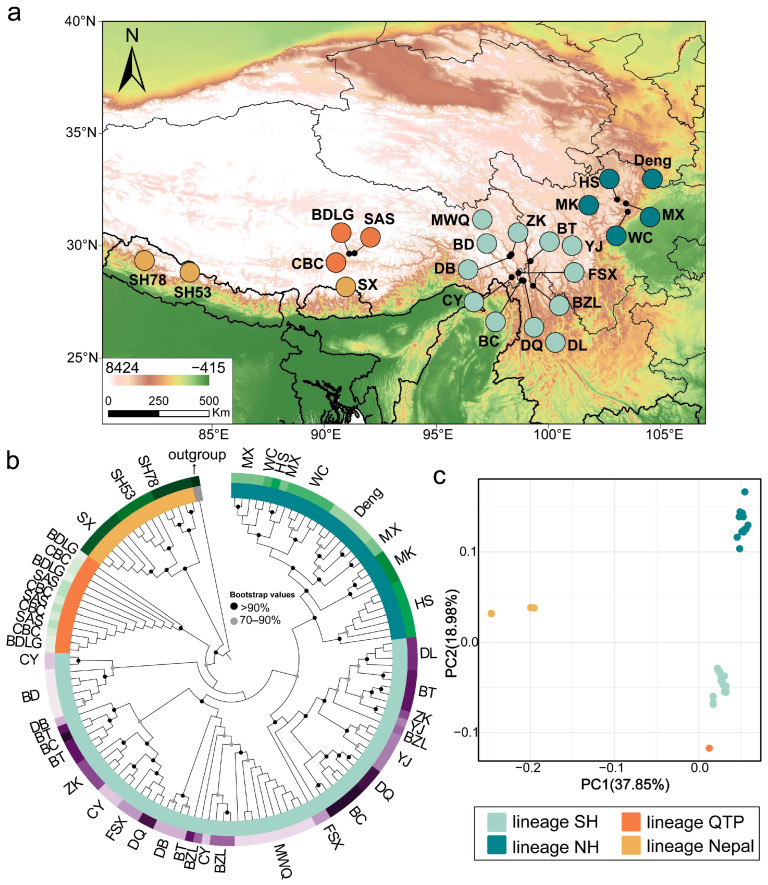
Population location and genetic clusters of *Mirabilis himalaica* based on phylogeny and PCA analyses. (**a**) Geographic distribution of 23 *M. himalaica* populations, with distinct colors representing the four genetic lineages. (**b**) Maximum likelihood (ML) phylogenetic tree of *M. himalaica* populations; only bootstrap support values (≥70%) are shown at nodes. (**c**) PCA plot of all individuals based on pan-plastome variants, with colors corresponding to the four genetic lineages identified in (**b**).

The ML-based phylogeny of 134 *M. himalaica* individuals revealed four well-supported genetic lineages, which geographically correspond to regions in Nepal (lineage Nepal), the Qinghai-Tibetan Plateau (lineage QTP), the southern Hengduan Mountains (hereafter lineage SH), and the northern Hengduan Mountains (hereafter lineage NH) (Figure 3a, b). Principal component analysis (PCA) further supported the four genetic lineages, with the first two principal components (PC1 and PC2) explaining 37.85% and 18.98% of the genetic variation, respectively. PC1 separated the lineage Nepal from other populations, whereas PC2 distinguished the lineage NH from lineages QTP and SH (Figure 3c). In contrast, population structure analysis showed partial inconsistency with the phylogenetic and PCA results, with incomplete lineage separation and admixture (Figure S2).

**Figure 4:**
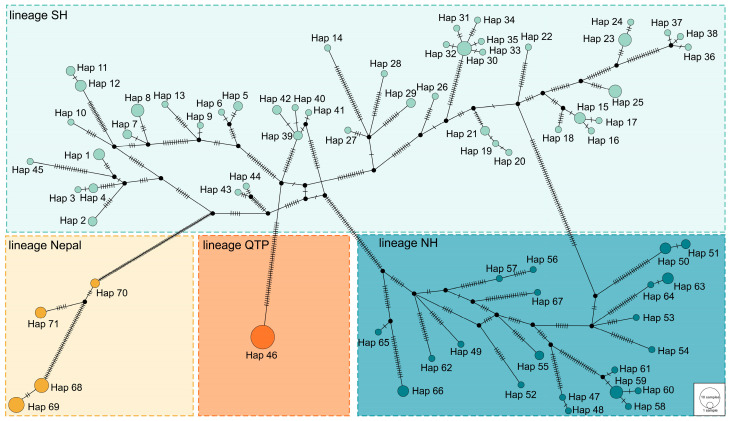
Haplotype network of the *Mirabilis himalaica* samples based on pan-plastome. The size of each pie is proportional to haplotype frequency, and colors indicate the four phylogenetic lineages.

A total of 71 plastome haplotypes were identified across all populations, which were grouped into four phylogroup lineages following haplotype network analysis (Figure 4 and Figure S3). Among these, lineage SH comprised Hap1–Hap45, while lineage QTP exclusively contained Hap46. The Hap47–Hap67 were detected in lineage NH, and Hap68–Hap71 were identified in lineage Nepal.

### 2.4. Genetic Diversity and Genetic Differentiation

Haplotype diversity was high overall (*H*_T_ = 0.985) compared to average within-population diversity (*H*_S_ = 0.580). The total *N*_ST_ (0.728) was significantly higher than *G*_ST_ (0.411, *p* < 0.05), indicating significant phylogeographical structure (Table S3). AMOVA revealed the majority of genetic variation (71.038%) among groups, with 17.41% among populations and 11.56% within populations (Table S6). Across all 23 populations, mean nucleotide diversity (*Pi*) was 0.00066, and haplotype diversity (*H*_d_) was 0.981 (Table S3). Among four lineages, lineage SH displayed the highest haplotype and nucleotide diversity (*H*_d_ = 0.982, *Pi* = 0.00033), while the lineage QTP exhibited the lowest diversity (*H*_d_ = 0.000, *Pi* = 0.00000) (Figure S4).

**Figure 5:**
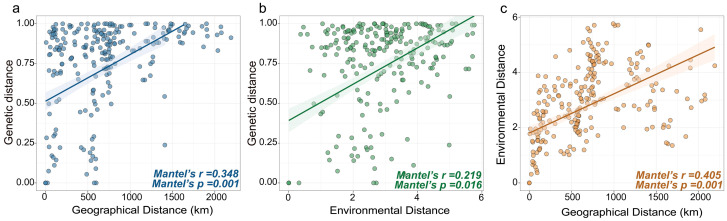
Mantel test correlations between phylogenetic, geographical, and environmental distances across *Mirabilis himalaica* populations. (**a**) Correlation between phylogenetic distance and geographical distance. (**b**) Correlation between phylogenetic distance and environmental distance. (**c**) Correlation between environmental distance and geographical distance. Each dot represents a pairwise comparison between populations. Solid lines indicate fitted linear regressions, and shaded areas represent 95% confidence intervals of the regression lines.

Pairwise *F*_ST_ values among groups ranged from 0.6096 to 0.8884, indicating strong genetic differentiation, and the highest divergence occurred between the lineages Nepal and QTP (*F*_ST_ = 0.888) (Figure S4). Mantel tests showed significant positive correlations between genetic distance and geographic distance (r = 0.348, *p* = 0.001), confirming the critical role of geographical isolation in shaping genetic differentiation (Figure 5). A significant correlation was also detected between genetic and environmental distances (r = 0.219, *p* = 0.016). Meanwhile, we detected a significant correlation between geographic and environmental distances (r = 0.405, *p* = 0.001).

### 2.5. Differential Metabolites and the Transcriptomic Regulation Across Altitude Gradients

**Figure 6:**
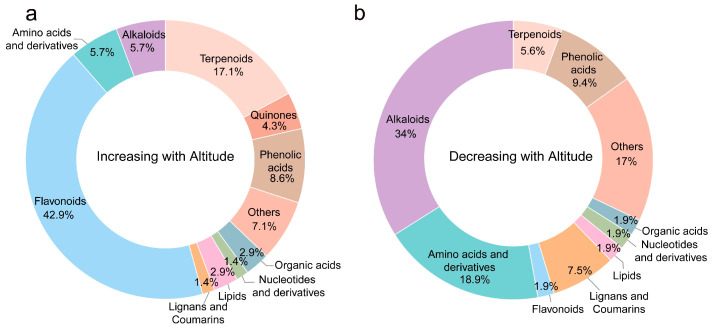
Distribution of differentially accumulated metabolites (DAMs) across major metabolite classes. (**a**) Metabolites with increasing abundance along the altitudinal gradient. (**b**) Metabolites with decreasing abundance along the altitudinal gradient.

A total of 2862 metabolites were detected in nine *M. himalaica* individuals across altitude gradients, which were classified into 13 categories, including flavonoids (19.5%), terpenoids (13%), amino acids and derivatives (12.1%), lipids (10.3%), alkaloids (10.1%), phenolic acids (9%), nucleotides and derivatives (4.1%), organic acids (3.8%), lignans and coumarins (3.6%), quinones (1%), tannins (0.5%), steroids (0.6%), and others (12.4%) (Figure S6). The PCA results showed a clear separation among the three altitude groups (Figure S7), and hierarchical clustering revealed different metabolic patterns across samples (Figure S8). To capture robust altitude-dependent trends, we applied a stringent intersection strategy based on pairwise comparisons among the three altitude groups (low vs. medium, medium vs. high, and low vs. high; Figure 6). Metabolites significantly upregulated in all three comparisons were defined as the increasing group (70 metabolites), while those significantly downregulated in all three comparisons formed the decreasing group (53 metabolites). Flavonoids were the most enriched class in the increasing group (42.9%), followed by terpenoids (17.1%). Conversely, alkaloids dominated the decreasing group (34%), with amino acids and derivatives ranking second (18.9%) (Figure 6). K-means clustering analysis of all expressed genes generated nine clusters (Figure S9). Cluster2 showed an upregulation trend with increasing altitude, and was mainly involved in cell cycle regulation, mitosis, chromosome segregation, and microtubule and cytoskeleton organization. Cluster1 gradually decreased with increasing altitude, and mainly enriched photosynthesis, light perception and signal transduction (Figure S10).

**Figure 7:**
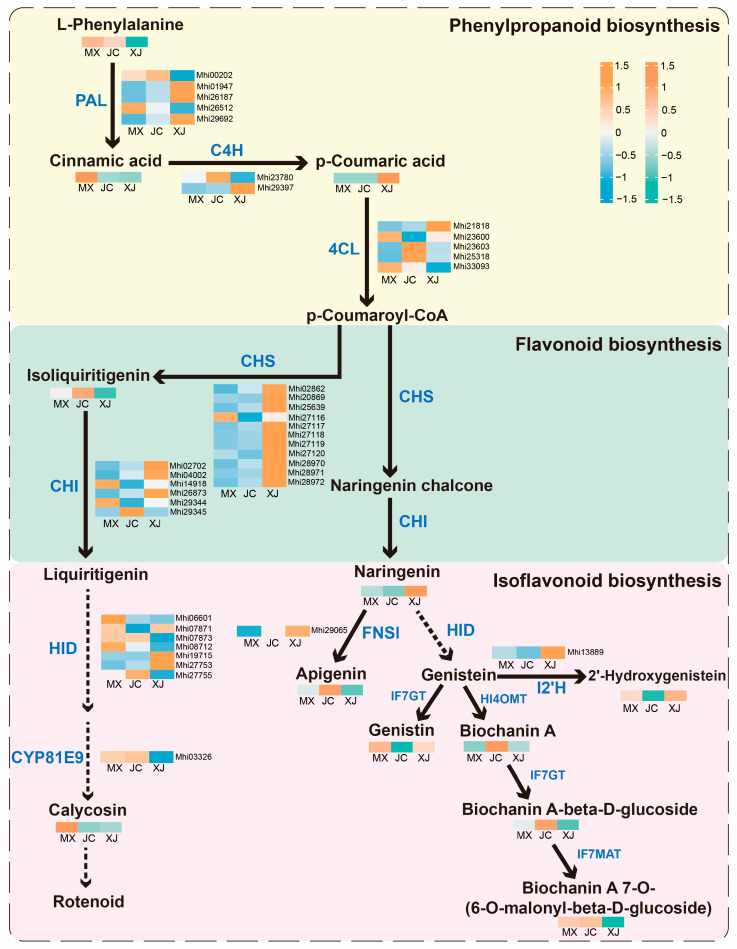
Integrated pathway map of flavonoid-related biosynthesis in *Mirabilis himalaica*. Gene expression and metabolite accumulation patterns are displayed along the altitudinal gradient. Transcript abundance and metabolite levels were normalized using Z-score transformation prior to visualization.

Given that flavonoids were the most abundant metabolites, we analyzed key enzymes and metabolites involved in the phenylalanine-derived flavonoid and isoflavonoid biosynthetic pathway. A total of 12 metabolites were identified in this biosynthetic pathway, including L-phenylalanine, cinnamic acid, isoliquiritigenin, naringenin, apigenin, genistein, and calycosin. Meanwhile, 39 genes encoding enzymes were identified, including phenylalanine ammonia-lyase (*PAL*), cinnamate 4-hydroxylase (*C4H*), chalcone synthase (*CHS*), chalcone isomerase (*CHI*), 2-hydroxyisoflavanone dehydratase (*HID*) and isoflavone 3′-hydroxylase (*CYP81E9*). We reconstructed the phenylalanine-related flavonoid and isoflavonoid biosynthetic pathway in *M. himalaica* (Figure 7). In this pathway, p-coumaroyl-CoA serves as a key metabolic precursor, which is further divided into two branches, leading to the formation of isoliquiritigenin and naringenin chalcone, respectively. Metabolites exhibited distinct accumulation patterns across different altitude groups of *M. himalaica*. For instance, p-Coumaric acid, naringenin, and genistin were mainly accumulated in the high-altitude group, whereas isoliquiritigenin, biochanin A, and apigenin were mainly enriched in the mid-altitude group, and cinnamic acid and claycosion showed higher accumulation in the low-altitude group. In addition, transcriptomic analysis revealed that key genes involved in flavonoid biosynthesis, such as *C4H*, *CHS*, *CHI* and *HID,* were differentially expressed among altitude groups and displayed expression patterns consistent with the accumulation of related flavonoid metabolites (Figure 7).

## 3. Discussion

The pan-plastomes of 134 *M. himalaica* individuals assembled in this study exhibited the same typical quadripartite structure as other angiosperm plants [29]. Annotation revealed 113 unique genes including 79 protein-coding genes, 30 tRNA genes, and four rRNA genes, consistent with the plastome architecture reported by Yuan et al. (2020) [30]. Owing to the highly conserved inheritance and structural stability of plastomes, plastid DNA fragments (e.g., *matK*, *rbcL*, and several intergenic regions) have been widely used in DNA barcoding for the authentication of medicinal plants and phylogenetic studies [18,31]. However, these several genes or intergenic loci often provide limited resolution for resolving relationships among closely related species [32,33,34]. Population-level pan-plastomes presented substantial variation including single-nucleotide variants and indels, which contributed to high-resolution identification in intraspecific identification and population differentiation [26,35]. In the present study, high numbers of genetic variations were shown across population-level pan-plastomes, including 620 SNVs, 171 indels, and four small inversions in *M. himalaica*, all of which are more abundant when compared to those other species like *Ulmus pumila* (313 SNVs and 277 indels) [25], *Adenocaulon himalaicum* (116 SNVs and 36 indels) [36] and *Distylium* (298 SNVs and 76 indels) [37]. Consistent with the pattern that non-coding regions generally show higher sequence variability, *M. himalaica* exhibited relatively high intraspecific pan-plastome variation compared with previously reported species. In addition, three mutation hotspots were identified, including *psbT–psbN*, *ccsA–ndhD*, and *ycf1* in *M. himalaica,* which were mainly located in the LSC and SSC regions. Compared to the LSC and SSC regions, the lower sequence divergence in IR regions is primarily due to homologous gene conversion between the two IR copies, which efficiently corrects newly arising mutations [38,39,40]. Additionally, IR regions typically encode highly conserved rRNAs under strong purifying selection, further suppressing variation [41,42]. Similar patterns have been reported in *Forsythia suspensa* [27], *Rosa* [43] and *Kaempferia* [44]. Therefore, these findings highlight the utility of pan-plastome sequences in examining genetic diversity and their role in the identification of Chinese medicinal herbs.

Analysis of selection pressure for all protein-coding genes indicated that the majority of genes exhibited dN/dS ratios below one, suggesting that they are primarily subject to purifying selection. This finding is highly consistent with the generally conserved plastomes and reflects the strong functional constraints acting on core genes involved in photosynthesis and transcription over long-term evolution [26,45]. Notably, *matK* and *rps4* showed dN/dS ratios greater than one, indicating that these genes may be under positive selection. Similar results have been detected in other studies of the Bambusoideae [46] and *Plantago* [18], suggesting that these genes may play a role in the adaptive evolution of plants. The *matK* encodes a maturase involved in group II intron splicing, which shows a relatively high evolutionary rate among plastid genes in angiosperms [47,48]. It is considered to play an important role in plastid transcript processing and gene expression regulation, which primarily acts on the *trnK* intron, and also participates in the splicing of introns from several other genes, such as *rps12*, *atpF*, and *rpoC1* [49,50]. This positive selection signal in *matK* may therefore reflect lineage-specific differential regulation of transcriptional efficiency. In addition, the *rps4* encodes the plastid ribosomal small subunit protein S4, which is involved in plastid ribosome assembly and protein translation [51,52]. Mutations in this gene have been shown to affect rRNA processing and plastid development [51,53]. Thus, positive selection acting on *rps4* may represent functional optimization of the plastid translation under specific environmental or developmental conditions. Furthermore, the *ndhD* gene was identified as a pseudogene in three Nepal populations, caused by premature termination of the coding sequence. The *ndhD* gene encodes a subunit of the plastid NAD(P)H dehydrogenase-like complex (NDH complex) that facilitates cyclic electron flow around photosystem I and photosynthetic efficiency under stress conditions [54,55], but it has been found to be dispensable for plant growth under optimal growth conditions [56]. The loss or pseudogenization of *ndh* genes has been reported in multiple lineages, including *Pedicularis* [57], *Gentiana* [58] and *Simmondsia* [59]. These patterns suggest that *ndh* gene loss in angiosperms is likely driven by relaxed selection and functional redundancy, particularly in lineages adapted to extreme environments, where shifts in photoprotection and electron transport pathways may reduce the dependence on *NDH* function [54,59,60]. However, the pseudogenization of *ndhD* is observed only in the Nepal lineage, whereas the QTP lineage with similarly extreme high-altitude environments retains a fully functional *ndhD* gene. This observation argues against the adaptive interpretation that pseudogenization confers a selective advantage under high-altitude conditions. Instead, neutral evolutionary forces offer more plausible explanations. The Nepal lineage represents a peripheral population at the southern edge of the species’ distribution, where smaller effective population sizes and geographic isolation could have facilitated the deleterious or neutral mutations by genetic drift or founder effects [61,62]. Therefore, the pseudogenization of *ndhD* in the Nepal lineage may be consistent with genetic drift or founder effects, rather than providing direct evidence of adaptive evolution based on the current data.

Our pan-plastome analysis revealed relatively low nucleotide diversity (*Pi* = 0.00066), but very high haplotype diversity (*H*_d_ = 0.981) in *M. himalaica.* This indicates the presence of numerous closely related haplotypes and is often associated with recent population expansion or rapid diversification following population bottlenecks [63]. Our “star-like” haplotype network also supported these results. Similar genetic patterns have also been reported in many alpine plants from the Himalaya–Hengduan Mountains (HHM), such as *Primula* [64], *Rhodiola* [65] and *Triosteum himalayanum* [66]. In addition, the population genetic structure of haplotype diversity (*H*_T_ = 0.985) greatly exceeded the average within-population diversity (*H*_S_ = 0.580), indicating that most genetic variation resides among, rather than within, populations [67,68]. This is a possible pattern of species with fragmented distributions and limited seed or pollen dispersal [69,70,71]. More importantly, the haplotype-based differentiation coefficient *N*_ST_ (0.728) was significantly higher than *G*_ST_ (0.411, *p* < 0.05), providing strong evidence of phylogeographic structure, suggesting an unusually strong influence of geographic barriers. The significant isolation-by-distance pattern (r = 0.348, *p* = 0.001) further demonstrated that gene flow is restricted with geographic distance. Based on the 620 SNVs and 171 indels identified from the pan-plastome, all 134 individuals from the 23 populations were consistently assigned to four distinct evolutionary lineages (lineages Nepal, QTP, SH and NH) using the phylogenetic reconstruction, genetic structure and haplotype network analysis. This assignment was strongly supported by high bootstrap values in the phylogeny, independent haplotypes from different lineages and high lineage differentiation (Figure 3 and Figure 4). Taken together, the complex topography of the HHM (such as deep river valleys and high mountain ridges) likely acts as biogeographic barrier to fragment populations and promote lineage divergence [72,73,74]. Second, historical climate oscillations during the Pleistocene would have repeatedly decreased alpine habitats into refugia, followed by expansion along elevational gradients and geographically separated haplotypes [74,75]. Given the high among-population differentiation, conservation units should be defined based on four genetically distinct evolutionary lineages (lineages Nepal, QTP, SH, NH). In addition, priority should be given to populations harboring unique or rare haplotypes to prevent irreversible loss of genetic resources in lineage Nepal and lineage QTP. Specifically, the following populations should be considered conservation priorities: (1) populations SH78 and SH53 in lineage Nepal and all populations from QTP containing private haplotypes not found elsewhere, and which, based on field observations, are subject to intensive harvesting pressure due to the medicinal value with limited individual numbers; (2) population MWQ from lineage SH that possesses the highest number of rare haplotypes distinct from others; (3) populations from lineage NH experiencing stronger human-induced disturbances due to growing along the roadside [76,77,78]. Further studies including demographic history, ecological vulnerability, and genetic load are required to evaluate the potential need for assisted migration [79,80]. We recommend targeted ex situ collections by systematically preserving seed or tissue samples from all major haplotype groups to secure a complete backup of the species’ genetic diversity [81].

The accumulation patterns of secondary metabolites along elevational gradients are central to understanding plant adaptation to environmental variation, yet the underlying regulatory mechanisms remain poorly understood [82,83]. High-elevation regions are characterized by harsh ecological conditions, including low temperatures, intense ultraviolet radiation, strong solar irradiance, and low oxygen availability [84,85]. These may enhance accumulation of specific secondary metabolites, thereby facilitating plant adaptation [86]. For example, leaf flavonoid content in *Ginkgo biloba* increased by over 150% along an elevational gradient, serving as antioxidant compounds to scavenge ROS [87]. Similarly, in *Nitraria* species high-altitude environments activate flavonoid biosynthetic pathways via ROS induction, upregulating key genes such as *C4H*, *F3H*, and *DFR* [82]. For *M. himalaica*, UV-B radiation has been shown to induce rotenoid biosynthesis, implicating ultraviolet exposure as a key environmental regulator of secondary metabolism in this species [88]. These findings suggest that high-elevation environmental conditions, particularly enhanced ultraviolet radiation, may be associated with the regulation of plant secondary metabolic pathways and the accumulation of bioactive compounds [89,90]. A total of 39 key genes related to phenylalanine-derived flavonoid and isoflavonoid pathways were identified, including phenylalanine ammonia-lyase (*PAL*), cinnamate 4-hydroxylase (*C4H*), chalcone synthase (*CHS*), and chalcone isomerase (*CHI*), 2-hydroxyisoflavanone dehydratase (*HID*) and isoflavone 3′-hydroxylase (*CYP81E9*). Notably, *CHS* and *CHI* act as key nodes linking phenylpropanoid metabolism to flavonoid formation. These genes showed a significantly upregulated expression pattern in high-elevation populations, suggesting altitudinal activation of this pathway under high-altitude conditions. Similar altitude-associated activation of flavonoid biosynthesis genes has also been reported in other alpine plants. For example, increased expression of *PAL*, *CHS* and *C4H* was observed in *Sinopodophyllum hexandrum* at higher elevations, accompanied by enhanced flavonoid accumulation and adaptation to strong light stress [91]. Similarly, coordinated increases in flavonoid accumulation and the expression of flavonoid biosynthesis genes, including *PAL*, *4CL*, *CHS*, *CHI*, and *F3′H*, were observed along elevational gradients in *Phlomoides rotata* [92]. Overall, the gene expression patterns in the flavonoid and isoflavonoid biosynthetic pathways of *M. himalaica* showed a general consistency with metabolite accumulation patterns across elevational groups. However, partial inconsistency between transcript levels and metabolite abundance was also observed for certain genes and compounds. This likely reflects the complex regulation of secondary metabolism. Importantly, these findings reveal altitude-associated patterns of gene expression and metabolite accumulation, but do not establish a direct mechanistic link between plastome variation and metabolic differentiation. Therefore, further functional validation, including gene overexpression, gene silencing, and enzyme activity assays, is required to clarify the precise regulatory roles of these candidate genes in flavonoid biosynthesis under high-altitude conditions in *M. himalaica*.

## 4. Materials and Methods

### 4.1. Plant Materials, DNA Sequencing and Plastome Assembly

Based on herbarium records, studies, and our previous field investigations, a total of 134 individuals of *Mirabilis himalaica* were collected from 21 populations in China and two populations in Nepal, covering its known distribution range (Table S7). For each population, 4–10 samples were collected and fresh leaves were dried using silica gel. Genomic DNA was extracted using the CTAB, following the method in the manufacturer’s instructions [93]. The quality of DNA was examined using the NanoPhotometer^®^ spectrophotometer ( Implen, Munich, Germany ) and 1% agarose gel electrophoresis. Paired-end libraries were prepared and high-throughput sequencing was subsequently performed on the DNBSEQ-T7 platform (MGI Tech Co., Ltd., Wuhan, China). Raw reads were filtered using fastp v0.23.4 with default parameters (Phred quality > 15, percent of unqualified bases <40) [94]. Plastomes were assembled from clean reads using GetOrganelle v1.7.7.1 [95] in embryophyte mode (-F embplant_pt) with a multi-k-mer scheme (-k 21, 45, 65, 85, 105) and 10 rounds of read extension (-R 10). The assembled plastomes were initially annotated using the online tool CPGAVAS2 [96] platform to annotate protein-coding genes, transfer RNA (tRNA) genes, and ribosomal RNA (rRNA) genes. The annotated plastomes were then manually checked for start and stop codons for each gene in Geneious Prime v2025.0.3, with the published plastomes of *Mirabilis himalaica* (MT535664) and the closely related species *Mirabilis jalapa* L. (MW894644) as references [30,97]. Finally, OGDRAW v1.3.1 was used to draw the physical map to visualize the plastomes with a default parameter [98].

To detect genes under potential selection, codon alignments of each protein-coding sequence were generated using MACSE v2.0 with default parameters [99]. A phylogenetic tree was constructed using IQ-TREE v2.2 under the GTR substitution model [100]. Based on the phylogenetic tree, the CODEML module in PAML v4.9 was used to estimate the non-synonymous substitution rate (dN), synonymous substitution rate (dS), and their ratio (dN/dS) for each protein-coding gene [101,102].

### 4.2. Genomic Variant Analysis from the Pan-Plastome

All 134 plastomes of *M. himalaica* were aligned using MAFFT v7.526 [103] with a default parameter, followed by manual check using Geneious Prime v2025.0.3. Genome size, gene content, and quadripartite structure were compared to characterize the pan-plastome evolution using Geneious Prime v2025.0.3. Single-nucleotide variants were identified using DnaSP v6 [104], and the counts and distribution patterns were calculated using a custom Python v 3.13.9 script. Furthermore, microstructural mutations in the *M. himalaica* plastome were classified into normal indels, repeat-related indels, microsatellite-related indels, and small inversions following Borsch and Quandt [105]. Normal indels are defined as insertions or deletions without recognizable repeat motifs or tandem structures. Microsatellite-related indels (SSRs) are insertions or deletions occurring in tandem repeats, typically composed of mono- or dinucleotide A/T-rich motifs. Repeat-related indels are short sequence motifs that are not classified as SSRs. Small inversions represent short, inverted segments capable of forming stem–loop secondary structures. Simple sequence repeats (SSRs) were detected using MISA v2.1 [106], with minimum repeat thresholds of ten repeat units for mononucleotides, six for dinucleotides, and five for tri- to hexanucleotide motifs. Long repeats (including forward, palindromic, reverse, and complementary) were identified with REPuter v2.74 [107] under default settings. The number, length, and positions of all microstructural mutations were recorded from whole-plastome alignments. Potentially informative characters (PICs) were summed by integrating nucleotide substitutions, indels, and small inversions collectively across plastomes [108].

### 4.3. Phylogenetic Analyses and Haplotype Network

Whole plastomes with only one single copy of the inverted repeat (IR) region were extracted and aligned using MAFFT v7.526 with default parameters [103]. The resulting alignment was used for Maximum likelihood (ML) phylogenetic reconstruction in IQ-TREE v2.2 [100], with *Mirabilis jalapa* L. as the outgroup. The best-fit nucleotide substitution model (K3Pu + F + I + R3) was automatically selected using ModelFinder in IQ-TREE v2.2. Branch support was evaluated using 1000 bootstrap replicates in combination with BNNI optimization. Population structure was inferred using fastSTRUCTURE v1.0 [109] based on SNVs with the optimal number of genetic clusters (K) ranging from 1 to 10. In addition, principal component analysis (PCA) was performed with GCTA v1.94.1 across populations [110].

Haplotype distribution was identified using DnaSP v6 and the distribution within each *M. himalaica* population was summarized [104]. A haplotype network was constructed using PopART v1.7 based on the TCS network method [111,112]. To identify the phylogroups, phylogenetic relationships among haplotypes were reconstructed using Bayesian Inference (BI) in MrBayes v3.2.7 [113]. The analysis was conducted using a Markov Chain Monte Carlo (MCMC) approach under the best-fit substitution model (GTR + I + G), which was selected through model testing in MrModeltest v2.4 implemented in PAUP v4.0 [114,115]. Four Markov chains were run for 10 million generations, with trees sampled every 1,000 generations. The first 25% of samples were discarded as burn-in, and convergence was assessed based on effective sample size (ESS) values calculated in TRACER v1.7.1 [116].

### 4.4. Genetic Diversity and Genetic Differentiation Analyses

The haplotype diversity (*H*_d_) and nucleotide diversity (*Pi*) were calculated using DnaSP v6 to assess genetic variation across *M. himalaica* populations and genetic lineages [104]. The overall haplotype diversity (*H*_T_) and average within-population diversity (*H*_S_) were calculated using PermutCpSSR v2.0 [117]. Population differentiation was analyzed by calculating gene differentiation coefficients *G*_ST_ and *N*_ST_, and the significance of phylogeographic structure tested using U-statistics based on 1000 permutations implemented in PermutCpSSR v2.0 [117]. An analysis of molecular variance (AMOVA) was performed using Arlequin v3.5.2 to examine the genetic variation within and among populations [118]. Pairwise genetic differentiation (*F*_ST_) between populations was calculated using Arlequin v3.5.2 [118]. To assess the effect of isolation by distance (IBD) and isolation by environment (IBE) on population genetic differentiation, Mantel tests were conducted to estimate the correlations between geographic and environmental distance matrices with population differentiation. The geographic distance matrix was estimated based on populations’ GPS coordinates using the ‘geosphere v1.5.20’ package in R [119]. For environmental distance, 19 bioclimatic variables were obtained from the WorldClim v2.1 database (Table S8) [120]. The relative contributions of variables were evaluated using MaxEnt v3.4.3 [121]. Based on the contribution ranking and correlations among variables (r < 0.75), five environmental variables were selected for subsequent analyses (Figure S5). These variables included isothermality (bio3), temperature annual range (bio7), mean temperature of wettest quarter (bio8), annual precipitation (bio12), and precipitation seasonality (bio15). Environmental distances were calculated using Euclidean distance derived from the first two principal components (Clim_PC1 and Clim_PC2) of the environmental variables [122]. Mantel was performed using the ‘vegan v2.6.10’ package in R with 999 permutations [123].

### 4.5. Data Analysis for Metabolomic and Transcriptomic Sequencing

To investigate metabolomic and transcriptomic variation along the altitudinal gradient while minimizing the confounding effects of genetic lineage background, three sampling sites were selected for metabolomic and transcriptomic analyses from the same genetic lineage (lineage NH in Results). Within this lineage, *M. himalaica* samples were collected from three representative sites along an elevational gradient: population MX (1812 m), population JC (2141 m), and population XJ (2636 m), with three biological replicates per population. Thus, the metabolomic and transcriptomic differences observed across these sites reflect altitude-associated variation rather than inter-lineage genetic divergence. Both metabolomic and transcriptomic analyses were performed by Metware Biotechnology (Wuhan, China). Metabolites were profiled using a UPLC–ESI–MS/MS platform and identified and relatively quantified based on database matching [124]. After data normalization, principal component analysis (PCA), clustering analysis, and differential metabolite screening were conducted [125,126], followed by KEGG pathway annotation [127]. Transcriptome sequencing was conducted on the Illumina platform, and clean reads were quality-filtered and mapped to the reference genome to estimate gene expression levels (FPKM) [128,129,130]. Differentially expressed genes were identified using DESeq2 and further subjected to Gene Ontology (GO) and KEGG enrichment analyses [127,129,131].

## Data Availability

The datasets generated and analyzed during this study have been deposited in the GenBank database.

## Supplementary Materials

The following supporting information can be downloaded at: https://www.mdpi.com/article/10.3390/plants15111691/s1. Figure S1: Representative plastome map of *Mirabilis himalaica.* Genes from different functional groups are colored in the outermost first ring. Figure S2: Population structure of *Mirabilis himalaica* inferred from STRUCTURE analysis. Each bar plot represents the estimated ancestry proportions of individuals for a given K value from 2 to 5. Figure S3: Bayesian phylogenetic tree reconstructed from cpDNA haplotypes of *Mirabilis himalaica*. Posterior probability (PP) values are shown at each node. Figure S4: Genetic diversity and differentiation among the four *Mirabilis himalaica* lineages (Nepal, QTP, SH, NH). Each bubble represents a lineage, and the numbers above the lines connecting two bubbles denote the pairwise *F*_ST_ values to measure genetic differentiation between lineages. Figure S5: Correlation heatmap of climatic factors used in *Mirabilis himalaica*. Circles in the upper triangular cells show pairwise correlations among climatic variables. Circle size is proportional to the absolute correlation coefficient. Red circles denote positive correlations, and blue circles denote negative correlations. Figure S6: Proportional distribution of metabolite classes based on compound counts. Figure S7: Principal component analysis (PCA) of metabolite profiles in *Mirabilis himalaica*. Figure S8: Hierarchical clustering heatmap of metabolite abundance based on Class I classification in *Mirabilis himalaica*. Figure S9: K-means clustering of all *Mirabilis himalaica* genes into 9 clusters. Figure S10: GO enrichment analysis was performed for genes in Cluster 1 and Cluster 2 of *Mirabilis himalaica*. Significantly enriched GO terms are shown according to the categories of biological process (BP), cellular component (CC), and molecular function (MF).; Table S1: Characteristics of quadripartite structure of the pan-plastome in 134 *Mirabilis himalaica* individuals. Table S2: All the 113 genes annotated from *Mirabilis himalaica* plastomes. Table S3: Statistics of genetic diversity and neutrality test results for all populations of *Mirabilis himalaica*. Table S4: Summary of PICs (SNVs, indels, and small inversions) and PIC ratio across coding and non-coding regions of the *Mirabilis himalaica* pan-plastome. Table S5: Small inversions in the *Mirabilis himalaica* pan-plastome. Table S6: Analysis of molecular variance (AMOVA) based on the pan-plastome sequences for *Mirabilis himalaica*. Table S7: The detailed formation for population characteristics and genetic diversity parameter of 23 *Mirabilis himalaica* populations analyzed in this study. Table S8: The contribution of 19 climatic variables to the distribution of *Mirabilis himalaica* and explanations of each climatic variable.
