# Supplementary material for: Pan-Plastome Evolution and Metabolite Variation Provide Insights to Conservation of the Tibetan Medicinal Plant Mirabilis himalaica
Source: Plants (Basel). 2026 May 30;15(11):1691. doi: 10.3390/plants15111691 (PMC13259483; doi:10.3390/plants15111691)
Supplement: Supplementary file 1 [file plants-15-01691-s001.zip › plants-4340661-supplementary.pdf]

SUPPLEMENTARY MATERIAL

Pan-plastome evolution and metabolite variation provide insights to conservation of the Tibetan medicinal plant *Mirabilis himalaica*

Yuxuan He[1,†], Nan Lin [1,2,3,*,†], Beier Duan[1], Jinhao Wang[1], Xiankun Wang[4], Zeyuan Cao[1] and Song Song [1,*]

[1] College of Life Science, Henan Agricultural University, Zhengzhou 450046, China; yuxuanhhee@outlook.com (Y.H.); 3137017707@qq.com (B.D.); wjh3295957935@163.com (J.W.); 19836155621@163.com (Z.C.)

[2] Henan Engineering Research Center for Osmanthus Germplasm Innovation and Resource Utilization, Henan Agricultural University, Zhengzhou 450046, China

[3] State Key Laboratory of Plant Diversity and Specialty Crops, Kunming Institute of Botany, Chinese Academy of Sciences, Kunming 650201, China

[4] College of Landscape Architecture, Henan Agricultural University, Zhengzhou 450046, China; wangxiankunx@163.com (X.W.)

* Correspondence: linnan@henau.edu.cn (N.L.); songsong@henau.edu.cn (S.S.)

† These authors contributed equally to this work.

Figure S1. Representative plastome map of *Mirabilis himalaica.* Genes from different functional groups are colored in the outermost first ring.

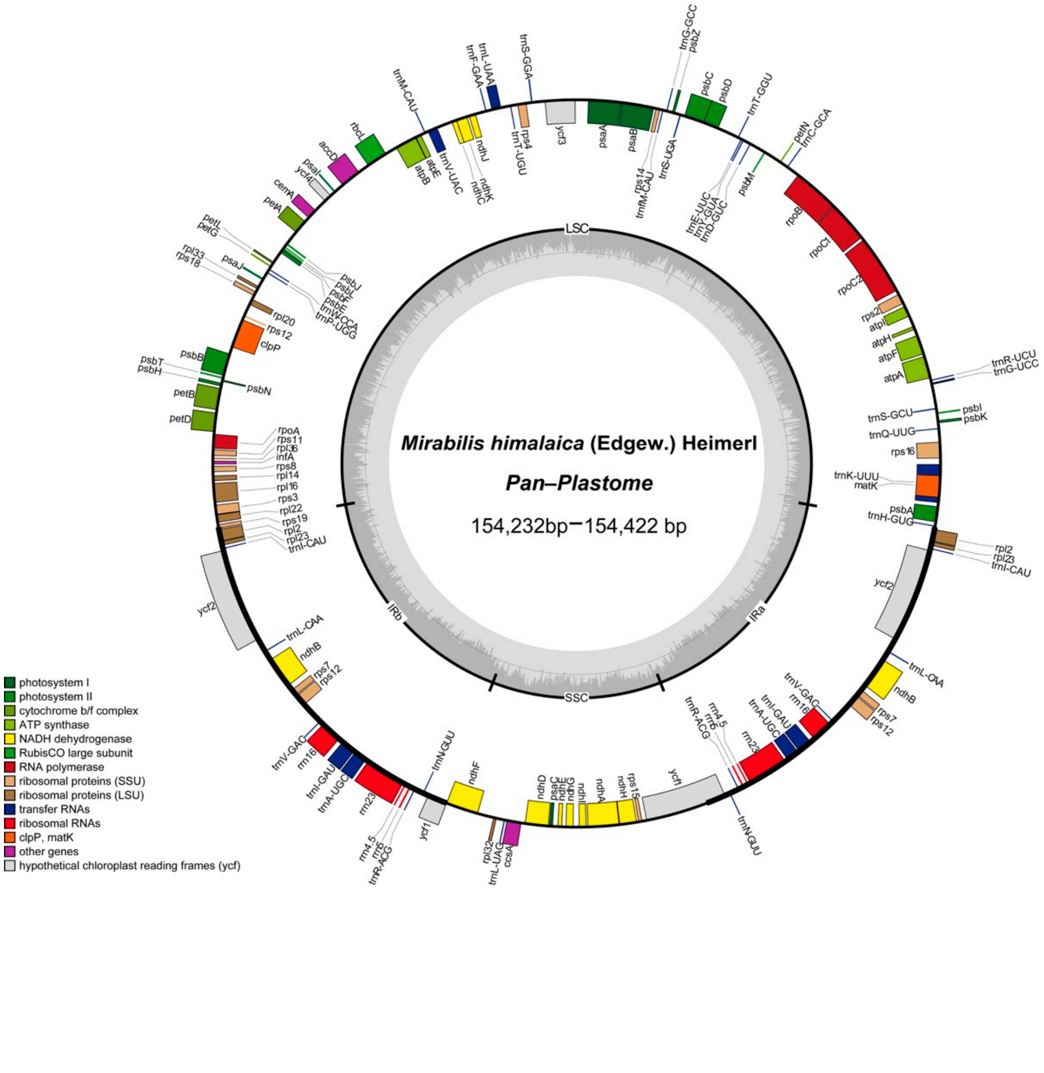

Figure S2 Population structure of *Mirabilis himalaica* inferred from STRUCTURE analysis. Each bar plot represents the estimated ancestry proportions of individuals for a given K value from 2 to 5.

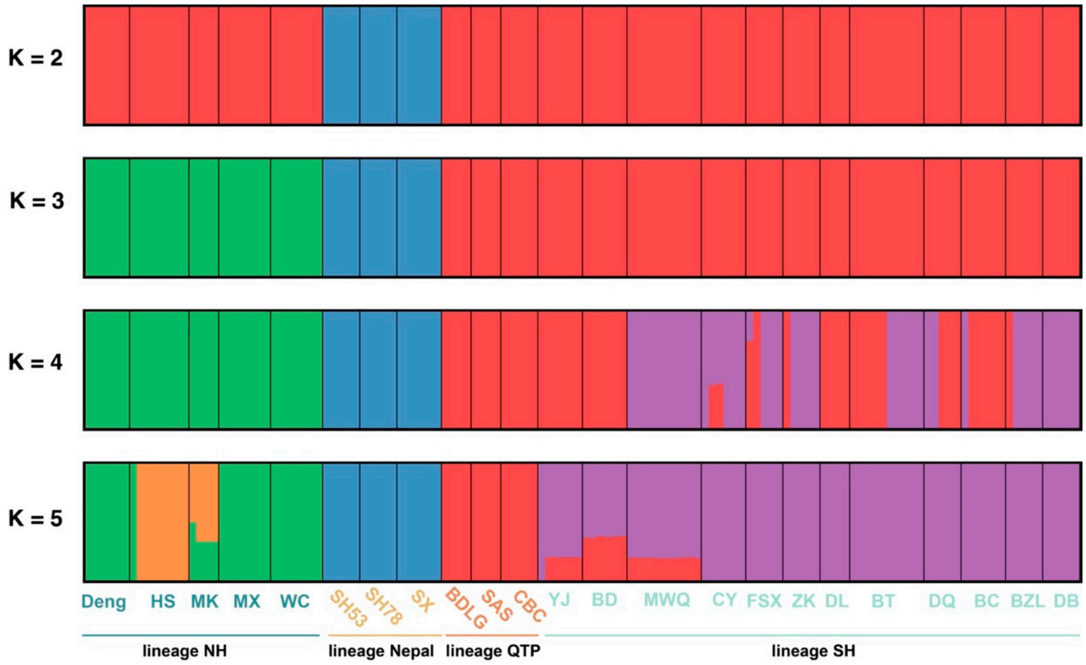

Figures S3. Bayesian phylogenetic tree reconstructed from cpDNA haplotypes of *Mirabilis himalaica*. Posterior probability (PP) values are shown at each node.

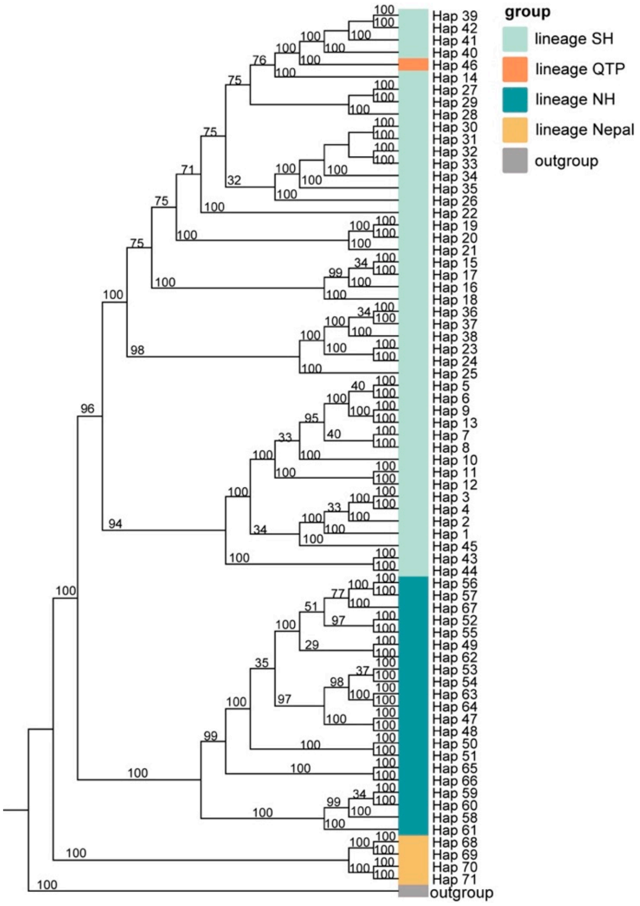

Figure S4. Genetic diversity and differentiation among the four *Mirabilis himalaica* lineages (Nepal, QTP, SH, NH). Each bubble represents a lineage, and the numbers above the lines connecting two bubbles denote the pairwise *F*st values to measure genetic differentiation between lineages.

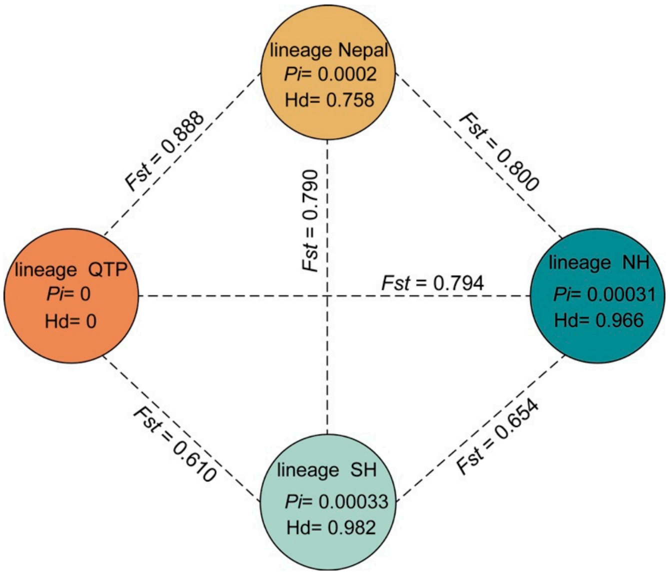

Figure S5 Correlation heatmap of climatic factors used in *Mirabilis himalaica*. Circles in the upper triangular cells show pairwise correlations among climatic variables. Circle size is proportional to the absolute correlation coefficient. Red circles denote positive correlations, and blue circles denote negative correlations.

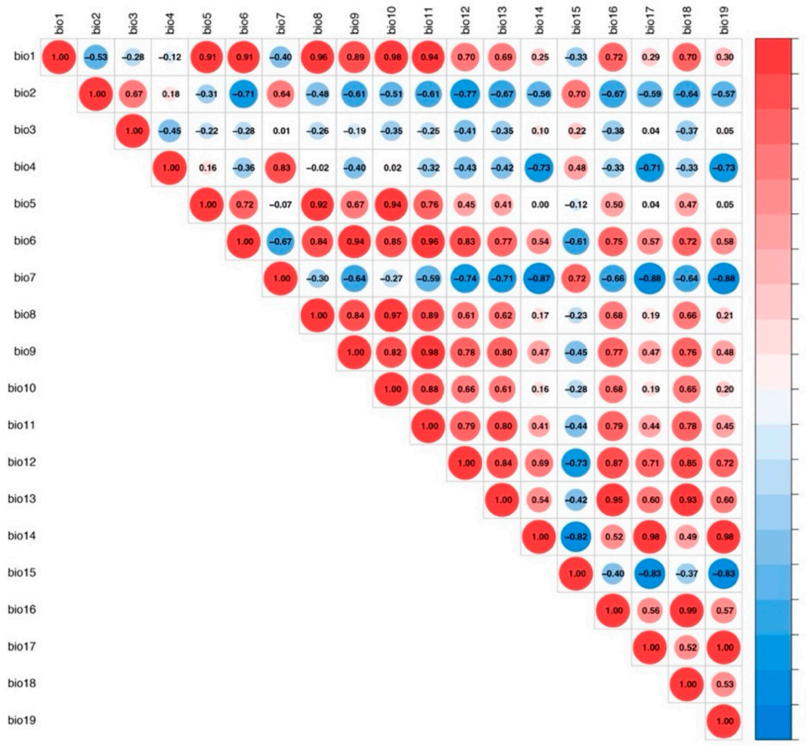

Figure S6 Proportional distribution of metabolite classes based on compound counts.

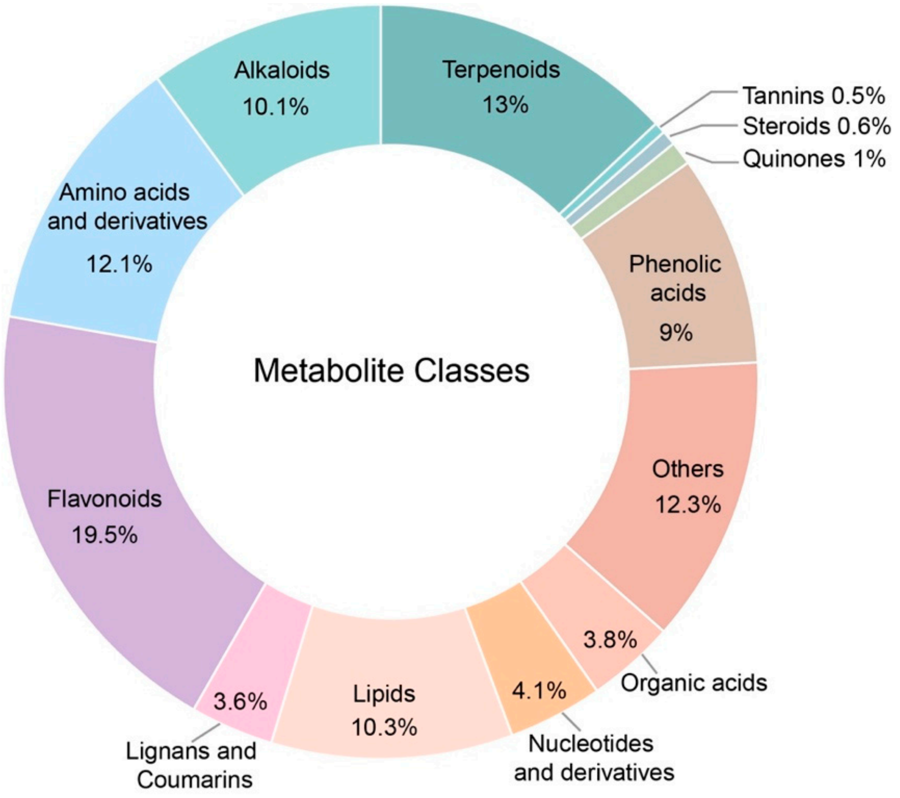

Figure S7 Principal component analysis (PCA) of metabolite profiles in *Mirabilis himalaica*.

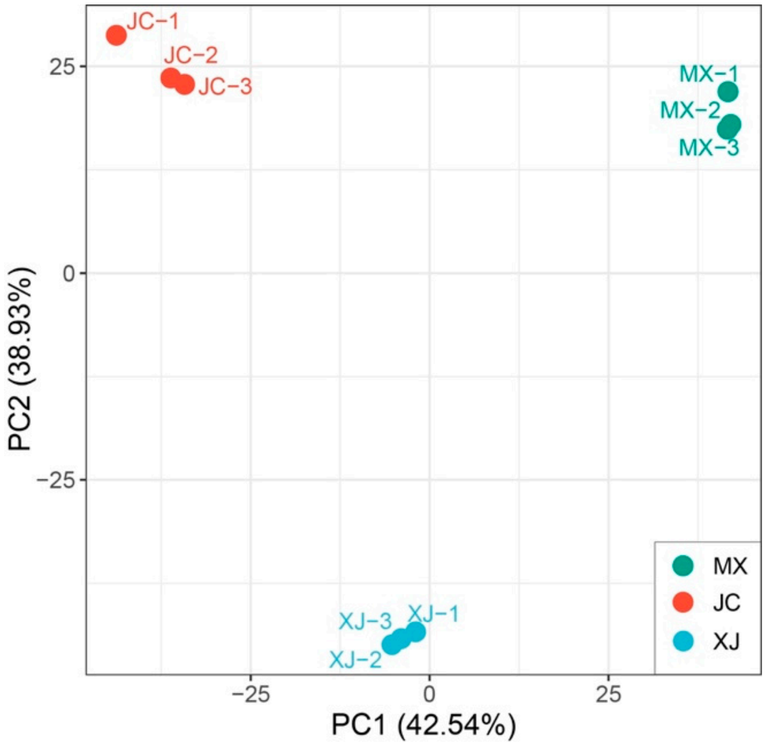

Figure S8 Hierarchical clustering heatmap of metabolite abundance based on Class I classification in *Mirabilis himalaica*.

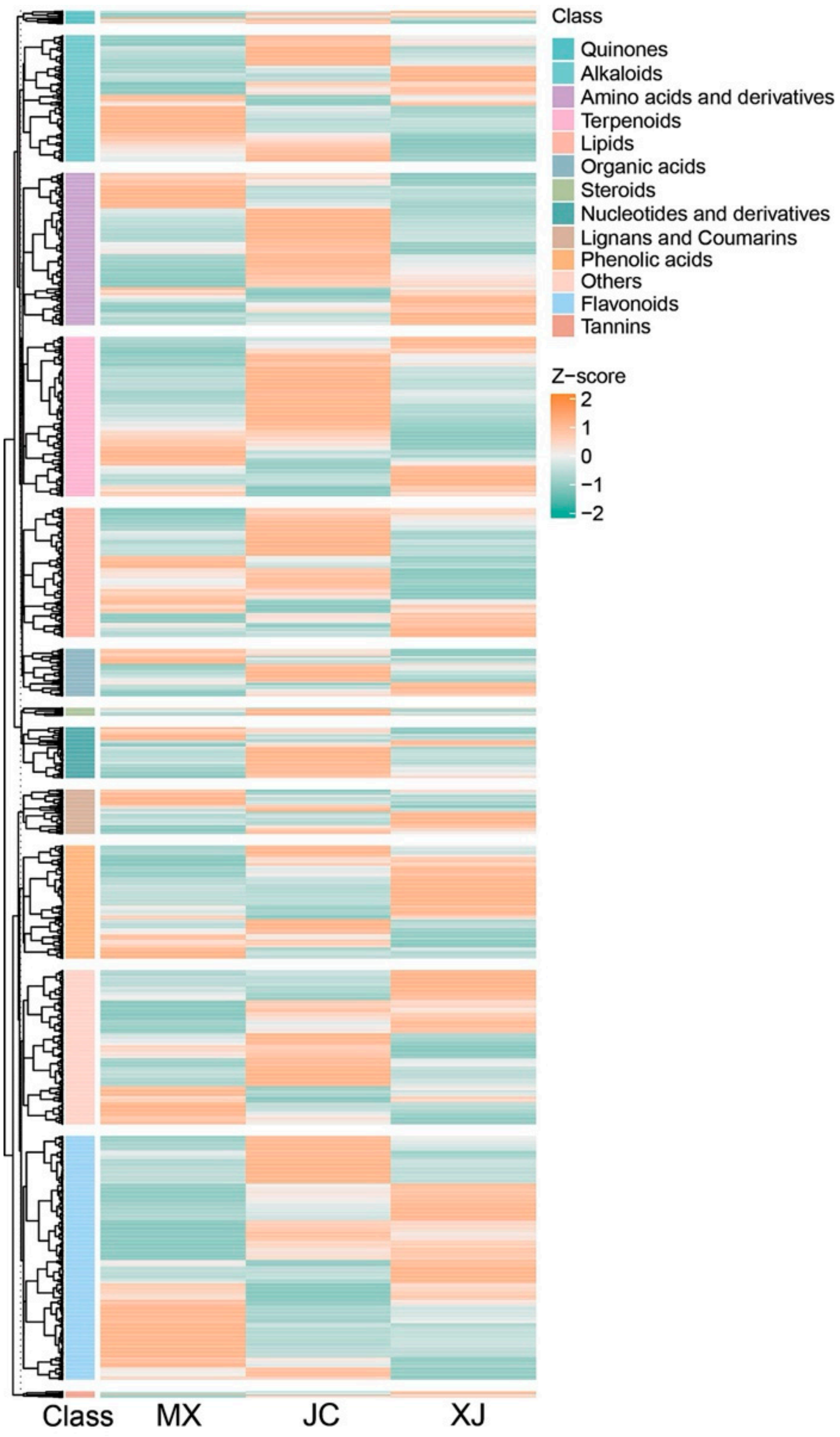

Figures S9 K-means clustering of all *Mirabilis himalaica* genes into 9 clusters.

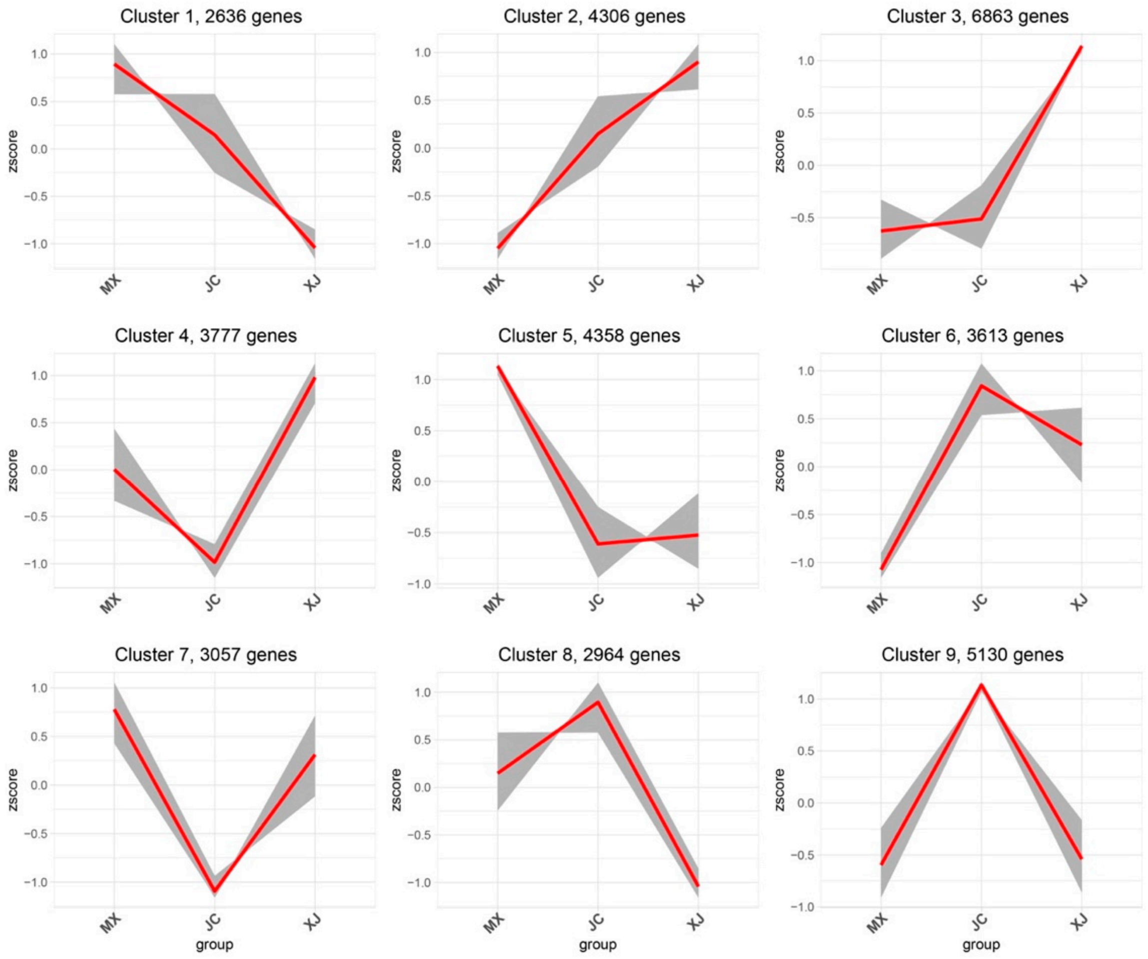

Figure S10 GO enrichment analysis was performed for genes in Cluster 1 and Cluster 2 of *Mirabilis himalaica*. Significantly enriched GO terms are shown according to the categories of biological process (BP), cellular component (CC), and molecular function (MF).

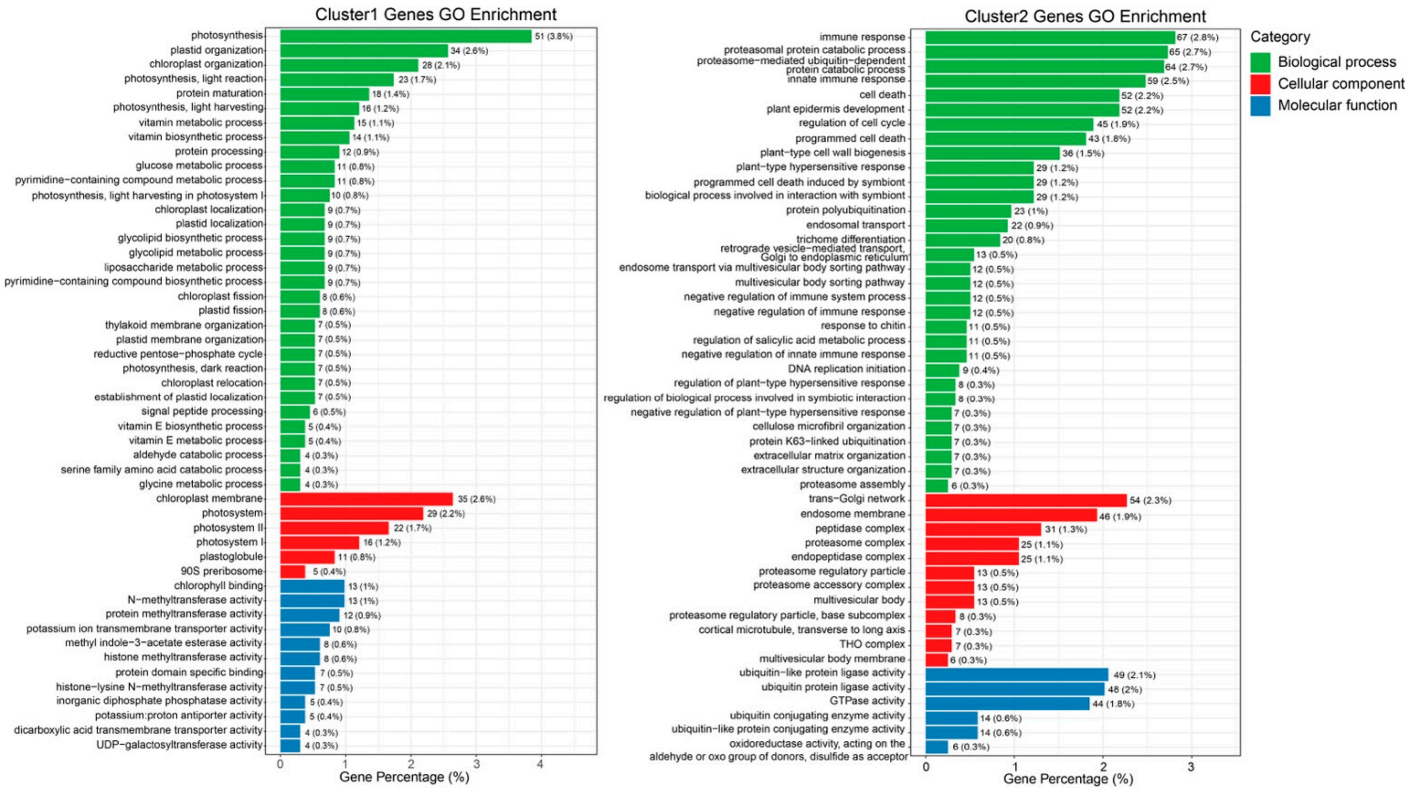

Table S1 Characteristics of quadripartite structure of the pan-plastome in 134 *Mirabilis himalaica* individual.

Sample	Population	LSC_Length (bp)	IR_Length (bp)	SSC_Length (bp)	total_Length (bp)
BC01	BC	85813	25299	17935	154346
BC03	BC	85813	25299	17935	154346
BC06	BC	85857	25299	17966	154421
BC08	BC	85830	25299	17945	154373
BC09	BC	85830	25299	17945	154373
BC10	BC	85813	25299	17935	154346
BD01	BD	85807	25299	17963	154368
BD03	BD	85807	25299	17963	154368
BD05	BD	85809	25299	17963	154370
BD07	BD	85807	25299	17963	154368
BD09	BD	85808	25299	17963	154369
BD10	BD	85807	25299	17963	154368
BDLG01	BDLG	85835	25299	17944	154377
BDLG03	BDLG	85835	25299	17944	154377
BDLG05	BDLG	85835	25299	17944	154377
BDLG06	BDLG	85835	25299	17944	154377
BT01	BT	85855	25299	17966	154419
BT02	BT	85858	25299	17966	154422

BT03	BT	85855	25299	17966	154419
BT04	BT	85814	25299	17929	154341
BT05	BT	85791	25299	17940	154329
BT06	BT	85855	25299	17966	154419
BT07	BT	85791	25299	17940	154329
BT08	BT	85791	25299	17940	154329
BT09	BT	85835	25299	17944	154377
BT10	BT	85835	25299	17944	154377
BZL01	BZL	85828	25299	17946	154372
BZL03	BZL	85827	25299	17947	154372
BZL06	BZL	85818	25299	17946	154362
BZL08	BZL	85819	25299	17946	154363
BZL10	BZL	85840	25299	17943	154381
CBC01	CBC	85835	25299	17944	154377
CBC02	CBC	85835	25299	17944	154377
CBC04	CBC	85835	25299	17944	154377
CBC05	CBC	85835	25299	17944	154377
CBC06	CBC	85835	25299	17944	154377
CY01	CY	85829	25299	17946	154373
CY03	CY	85824	25299	17948	154370
CY05	CY	85826	25299	17949	154373
CY07	CY	85833	25299	17928	154359
CY09	CY	85824	25299	17948	154370
CY10	CY	85851	25299	17929	154378
DB01	DB	85828	25299	17946	154372
DB03	DB	85828	25299	17946	154372
DB06	DB	85857	25299	17946	154401
DB08	DB	85829	25299	17946	154373
DB10	DB	85830	25299	17946	154374
Deng01	Deng	85775	25299	17906	154279
Deng02	Deng	85779	25295	17911	154280
Deng03	Deng	85779	25295	17911	154280
Deng04	Deng	85779	25295	17911	154280
Deng05	Deng	85779	25295	17911	154280
Deng06	Deng	85776	25299	17906	154280
DL01	DL	85838	25299	17945	154381
DL02	DL	85838	25299	17945	154381
DL03	DL	85838	25299	17945	154381
DL04	DL	85838	25299	17945	154381
DQ01	DQ	85835	25299	17945	154378
DQ03	DQ	85827	25299	17948	154373
DQ05	DQ	85836	25299	17945	154379
DQ07	DQ	85826	25299	17948	154372

DQ08	DQ	85836	25299	17945	154379
FSX01	FSX	85825	25299	17948	154371
FSX03	FSX	85845	25299	17944	154387
FSX05	FSX	85825	25299	17948	154371
FSX06	FSX	85825	25299	17948	154371
FSX07	FSX	85847	25298	17926	154369
HS01	HS	85735	25299	17905	154238
HS02	HS	85735	25299	17905	154238
HS03	HS	85735	25299	17905	154238
HS04	HS	85735	25299	17905	154238
HS05	HS	85735	25299	17905	154238
HS06	HS	85735	25299	17905	154238
HS07	HS	85771	25299	17905	154274
HS08	HS	85735	25299	17905	154238
MK01	MK	85777	25299	17896	154271
MK02	MK	85767	25299	17906	154271
MK03	MK	85767	25299	17906	154271
MK04	MK	85767	25299	17906	154271
MWQ01	MWQ	85836	25299	17946	154380
MWQ02	MWQ	85837	25299	17946	154381
MWQ03	MWQ	85837	25299	17946	154381
MWQ04	MWQ	85837	25299	17946	154381
MWQ05	MWQ	85836	25299	17946	154380
MWQ06	MWQ	85837	25299	17946	154381
MWQ07	MWQ	85837	25299	17946	154381
MWQ08	MWQ	85836	25299	17946	154380
MWQ09	MWQ	85837	25299	17946	154381
MWQ10	MWQ	85836	25299	17946	154380
MX01	MX	85773	25299	17905	154276
MX02	MX	85733	25299	17901	154232
MX03	MX	85781	25299	17905	154284
MX04	MX	85792	25299	17913	154303
MX05	MX	85787	25306	17896	154295
MX06	MX	85787	25306	17896	154295
MX07	MX	85777	25299	17906	154281
SAS01	SAS	85835	25299	17944	154377
SAS03	SAS	85835	25299	17944	154377
SAS05	SAS	85835	25299	17944	154377
SAS07	SAS	85835	25299	17944	154377
SH53_1	SH53	85791	25299	17949	154338
SH53_4	SH53	85791	25299	17949	154338
SH53_5	SH53	85791	25299	17949	154338
SH53_7	SH53	85791	25299	17949	154338

SH53_9	SH53	85791	25299	17949	154338
SH78_1	SH78	85734	25299	17948	154280
SH78_10	SH78	85746	25299	17948	154292
SH78_2	SH78	85734	25299	17948	154280
SH78_7	SH78	85746	25299	17948	154292
SH78_8	SH78	85746	25299	17948	154292
SX01	SX	85786	25299	17949	154333
SX03	SX	85786	25299	17949	154333
SX05	SX	85786	25299	17949	154333
SX07	SX	85786	25299	17949	154333
SX09	SX	85786	25299	17949	154333
SX10	SX	85786	25299	17949	154333
WC01	WC	85821	25293	17913	154320
WC02	WC	85758	25299	17905	154261
WC03	WC	85755	25299	17905	154258
WC04	WC	85755	25299	17905	154258
WC06	WC	85755	25299	17905	154258
WC07	WC	85755	25299	17905	154258
WC08	WC	85755	25299	17905	154258
YJ001	YJ	85833	25299	17944	154375
YJ002	YJ	85833	25299	17944	154375
YJ003	YJ	85845	25299	17953	154396
YJ004	YJ	85833	25299	17944	154375
YJ005	YJ	85833	25299	17944	154375
YJ006	YJ	85833	25299	17944	154375
ZK01	ZK	85840	25299	17944	154382
ZK03	ZK	85828	25299	17948	154374
ZK05	ZK	85828	25299	17948	154374
ZK06	ZK	85828	25299	17948	154374
ZK07	ZK	85828	25299	17948	154374

Table S2 All the 113 genes annotated from *Mirabilis himalaica* plastomes.

Gene Category	Functional Group	Gene Name
Protein-coding Genes	Photosystem I	*psaA, psaB, psaC, psaI, psaJ*
	Photosystem II	*psbA, psbB, psbC, psbD, psbE, psbF, psbH, psbI, psbJ, psbK, psbT, psbL, psbZ, psbM, psbN*
	Small subunit of ribosome	*rps2, rps3, rps4, rps7, rps8, rps11, rps12*, rps14, rps15, rps16, rps18, rps19*
	Large subunit of ribosome	*rpl2, rpl14, rpl16, rpl20, rpl22, rpl23, rpl32, rpl33, rpl36*
	NADH dehydrogenase	*ndhA, ndhB, ndhC, ndhD, ndhE, ndhF, ndhG, ndhH, ndhI, ndhJ, ndhK*
	Cytochrome b/f complex	*PetA, petB, petD, petG, petL, petN*
	ATP synthase	*atpA, atpB, atpE, atpF, atpH, atpI*
	RNA polymerase	*rpoA, rpoB, rpoC1, rpoC2*
	Large subunit of Rubisco	*rbcL*
	Unknown function	*ycf1, ycf2, ycf3*, ycf4*
	Cytochrome c biogenesis protein	*ccsA*
	Envelope membrane protein	*cemA*
	Subunit of ATP-dependent Clp	*clpP**
	Translation initiation factor	*infA*
	Subunit of acetyl-CoA	*accD*
	Maturase	*matK*
tRNA genes	Transfer RNA	*trnA-UGC, trnC-GCA, trnD-GUC, trnE-UUC, trnF-GAA, trnG-UCC, trnG-GCC, trnH-GUG, trnI-CAU, trnI-GAU, trnK-UUU, trnL-CAA, trnL-UAA, trnL-UAG, trnM-CAU, trnN-GUU, trnP-UGG, trnQ-UUG, trnR-ACG, trnR-UCU, trnS-GGA, trnS-GCU, trnS-UGA, trnT-GGU, trnT-UGU, trnV-GAC, trnV-UAC, trnW-CCA, trnY-GUA, trnfM-CAU*
rRNA genes	Ribosomal RNA	*rrna 4.5, rrna 5, rrna 16, rrna 23*

Table S3 Statistics of genetic diversity and neutrality test results for all populations of *Mirabilis himalaica.*

Parameters	Value
Number of haplotypes	71
Haplotype diversity	0.981
Pi	0.00066
Parsimony informative sites	468
Singleton sites	152
H_T	0.985
H_S	0.580
N_{ST} / G_{ST} (pvalue)	0.728/0.411 ($p<0.05$)

Table S4 Summary of PICs (SNVs, indels, and small inversions) and PIC ratio across coding and non-coding regions of the *Mirabilis himalaica* pan-plastome.

**Number**	Interval	Length	Type	SNV count	Indel count	Small inversions	PICs	PICs ratio%
1	*psbA*	1062	gene	1	0	0	1	0.094
2	*matK*	1518	gene	6	1	0	7	0.461
3	*rps16*	1146	gene	5	1	0	6	0.524
4	*atpA*	1524	gene	1	0	0	1	0.066
5	*atpF*	1347	gene	5	2	0	7	0.520
6	*atpH*	246	gene	1	0	0	1	0.407
7	*atpI*	744	gene	1	0	0	1	0.134
8	*rps2*	711	gene	1	0	0	1	0.141
9	*rpoC2*	4119	gene	13	0	0	13	0.316
10	*rpoC1*	2805	gene	9	3	0	12	0.428
11	*rpoB*	3219	gene	9	0	0	9	0.280
12	*psbC*	1422	gene	1	0	0	1	0.070
13	*rps14*	303	gene	2	0	0	2	0.660
14	*psaA*	2253	gene	4	0	0	4	0.178
15	*ycf3*	2066	gene	5	1	0	6	0.290

16	*rps4*	606	gene	4	0	0	4	0.660
17	*ndhJ*	477	gene	1	0	0	1	0.210
18	*ndhK*	768	gene	2	0	0	2	0.260
19	*ndhC*	363	gene	1	0	0	1	0.275
20	*atpE*	402	gene	2	0	0	2	0.498
21	*atpB*	1497	gene	3	0	0	3	0.200
22	*rbcL*	1446	gene	2	0	0	2	0.138
23	*accD*	1415	gene	11	3	0	14	0.989
24	*ycf4*	555	gene	1	0	0	1	0.180
25	*cemA*	699	gene	2	0	0	2	0.286
26	*petA*	963	gene	4	0	0	4	0.415
27	*psbL*	81	gene	1	0	0	1	1.235
28	*psbF*	120	gene	1	0	0	1	0.833
29	*psbE*	252	gene	1	0	0	1	0.397
30	*petL*	132	gene	0	2	0	2	1.515
31	*petG*	114	gene	1	0	0	1	0.877
32	*rpl33*	207	gene	1	0	0	1	0.483
33	*rpl20*	414	gene	3	0	0	3	0.725
34	*clpP*	2009	gene	10	7	0	17	0.846
35	*psbB*	1527	gene	1	0	0	1	0.065
36	*petB*	1468	gene	4	2	0	6	0.409
37	*petD*	1289	gene	9	4	0	13	1.009
38	*rpoA*	1017	gene	4	0	0	4	0.393
39	*rps11*	417	gene	5	0	0	5	1.199
40	*rps8*	405	gene	3	0	0	3	0.741
41	*rpl16*	1330	gene	4	3	0	7	0.526

42	*rps3*	657	gene	4	0	0	4	0.609
43	*rpl22*	546	gene	3	1	0	4	0.733
44	*rps19*	279	gene	3	0	0	3	1.075
45	*rpl2*	825	gene	1	0	0	1	0.121
46	*ycf2*	6471	gene	3	0	0	3	0.046
47	*ndhB*	2147	gene	2	0	0	2	0.093
48	*ycf1*	1371	gene	61	1	0	62	4.522
49	*ndhF*	2232	gene	12	0	0	12	0.538
50	*rpl32*	225	gene	1	0	0	1	0.444
51	*ccsA*	993	gene	13	2	0	15	1.511
52	*ndhD*	1617	gene	8	0	0	8	0.495
53	*ndhE*	306	gene	2	0	0	2	0.654
54	*ndhG*	531	gene	2	0	0	2	0.377
55	*ndhI*	513	gene	2	0	0	2	0.390
56	*ndhA*	2164	gene	9	0	0	9	0.416
57	*ndhH*	1182	gene	3	0	0	3	0.254
58	*rps15*	273	gene	1	0	0	1	0.366
1	*trnH-GUG-psbA*	236	intergenic	7	1	0	8	3.390
2	*psbA-matK*	603	intergenic	3	1	0	4	0.663
3	*matK-rps16*	1148	intergenic	10	4	0	14	1.220
4	*rps16-psbK*	1270	intergenic	12	3	0	15	1.181
5	*psbK-psbI*	434	intergenic	5	2	0	7	1.613
6	*psbI-atpA*	2367	intergenic	26	15	0	41	1.732
7	*atpA-atpF*	146	intergenic	0	2	0	2	1.370
8	*atpF-atpH*	416	intergenic	4	1	0	5	1.202
9	*atpH-atpI*	722	intergenic	4	1	0	5	0.693

10	*atpI-rps2*	207	intergenic	2	0	0	2	0.966
11	*rps2-rpoC2*	265	intergenic	5	1	0	6	2.264
12	*rpoC2-rpoC1*	175	intergenic	2	0	0	2	1.143
13	*rpoB-petN*	1429	intergenic	8	2	0	10	0.700
14	*petN-psbM*	1144	intergenic	10	4	1	15	1.311
15	*psbM-psbD*	3492	intergenic	24	14	0	38	1.088
16	*psbC-psbZ*	673	intergenic	3	1	0	4	0.594
17	*psbZ-rps14*	828	intergenic	3	2	0	5	0.604
18	*psaA-ycf3*	862	intergenic	9	1	0	10	1.160
19	*ycf3-rps4*	1259	intergenic	5	3	0	8	0.635
20	*rps4-ndhJ*	2900	intergenic	18	9	0	27	0.931
21	*ndhJ-ndhK*	127	intergenic	1	0	0	1	0.787
22	*ndhC-atpE*	2243	intergenic	20	7	0	27	1.204
23	*atpB-rbcL*	792	intergenic	6	2	0	8	1.010
24	*rbcL-accD*	759	intergenic	6	3	0	9	1.186
25	*accD-psaI*	638	intergenic	5	6	0	11	1.724
26	*psaI-ycf4*	312	intergenic	2	2	0	4	1.282
27	*ycf4-cemA*	880	intergenic	4	3	0	7	0.795
28	*cemA-petA*	326	intergenic	1	2	0	3	0.920
29	*petA-psbJ*	938	intergenic	17	4	1	22	2.345
30	*psbE-petL*	783	intergenic	3	3	0	6	0.766
31	*petG-psaJ*	882	intergenic	4	5	0	9	1.020
32	*psaJ-rpl33*	500	intergenic	1	2	0	3	0.600
33	*rpl33-rps18*	197	intergenic	4	1	0	5	2.538
34	*rps18-rpl20*	250	intergenic	2	1	0	3	1.200
35	*rpl20-rps12*	791	intergenic	4	3	0	7	0.885

36	*rps12-clpP*	202	intergenic	2	2	0	4	1.980
37	*clpP-psbB*	462	intergenic	3	2	0	5	1.082
38	*psbT-psbN*	60	intergenic	6	0	0	6	10.000
39	*psbH-petB*	150	intergenic	1	1	0	2	1.333
40	*petB-petD*	207	intergenic	1	1	0	2	0.966
41	*petD-rpoA*	144	intergenic	0	1	0	1	0.694
42	*rpoA-rps11*	71	intergenic	1	0	0	1	1.408
43	*rpl36-infA*	105	intergenic	1	0	0	1	0.952
44	*infA-rps8*	118	intergenic	3	0	0	3	2.542
45	*rps8-rpl14*	243	intergenic	5	0	0	5	2.058
46	*rpl14-rpl16*	142	intergenic	1	2	0	3	2.113
47	*rpl16-rps3*	151	intergenic	3	2	0	5	3.311
48	*rpl22-rps19*	122	intergenic	2	1	0	3	2.459
49	*rpl23-ycf2*	327	intergenic	1	0	0	1	0.306
50	*ycf2-ndhB*	1594	intergenic	2	0	0	2	0.125
51	*rps12-ycf1*	10432	intergenic	18	4	0	22	0.211
52	*ndhF-rpl32*	1092	intergenic	13	7	0	20	1.832
53	*rpl32-ccsA*	697	intergenic	9	2	0	11	1.578
54	*ccsA-ndhD*	239	intergenic	13	0	1	14	5.858
55	*psaC-ndhE*	325	intergenic	2	1	0	3	0.923
56	*ndhE-ndhG*	243	intergenic	1	1	0	2	0.823
57	*ndhG-ndhI*	359	intergenic	3	0	0	3	0.836
58	*ndhI-ndhA*	79	intergenic	3	0	0	3	3.797
59	*ndhH-rps15*	105	intergenic	2	0	0	2	1.905
60	*rps15-ycf1*	275	intergenic	9	0	1	10	3.636

Table S5 Small inversions in the *Mirabilis himalaica* pan-plastome.

Regions	Position	Location	Length (bp)	
			Loop	Stem
LSC	*petN-psbM*	space	4	20
LSC	*petA-psbJ*	space	18	34
SSC	*ccsA-ndhD*	space	17	17
SSC	*rps15-ycf1*	space	7	20

Table S6 Analysis of molecular variance (AMOVA) based on the pan-plastome sequences for *Mirabilis himalaica*.

Source of variation	Sum of squares	Variance components	Percentage of variation	Fixation indices
Among groups	11198.594	127.22208	71.03788	F_{CT} = 0.60096 (p<0.01)
Within groups	3850.392	31.17095	17.40514	F_{SC} = 0.88443 (p<0.01)
Within populations	2297.417	20.69745	11.55698	F_{ST} = 0.71038 (p<0.01)

Table S7 The detailed formation for population characteristics and genetic diversity parameter of 23 *Mirabilis himalaica* populations analyzed in this study.

**Population code**	**Number of individuals**	**Latitude/ Longitude**	**Pi*10^{-3}**	**Hd**	**Haplotypes**
BC	6 (BC01/BC03/BC06/BC08/BC09/BC10)	28.46N,98.81E	0.120	0.733	Hap1(3), Hap2(2), Hap15(1)
BD	6 (BD01/BD03/BD05/BD07/BD09/BD10)	30.08N,97.29E	0.010	0.867	Hap39(2), Hap40(1), Hap41(1), Hap42(2)
BDLG	4 (BDLG01/BDLG03/BDLG05/BDLG06)	29.65N,91.11E	0.000	0.000	Hap46(4)
BT	10 (BT01-BT10)	29.32N,99.20E	0.260	0.933	Hap5(2), Hap6(1), Hap7(2), Hap15(2),

					Hap16(1), Hap17(1), Hap22(1)
BZL	5 (BZL01/BZL03/BZL06/BZL08/BZL10)	28.22N,99.31E	0.240	0.900	Hap10(1), Hap26(1), Hap27(1), Hap29(2)
CBC	5 (CBC01/CBC02/CBC04/CBC05/CBC06)	29.25N,90.52E	0.000	0.000	Hap46(5)
CY	6 (CY01/CY03/CY05/CY07/CY09/CY10)	28.60N,98.35E	0.330	1.000	Hap28(1), Hap36(1), Hap37(1), Hap38(1), Hap43(1), Hap44(1)
DB	5 (DB01/DB03/DB06/DB08/DB10)	29.54N,98.27E	0.070	0.900	Hap18(1), Hap19(1), Hap20(1), Hap21(2)
Deng	6 (Deng01-Deng06)	32.95N,104.64E	0.160	0.800	Hap47(1), Hap48(1), Hap63(3), Hap64(1)
DL	4 (DL01- DL04)	25.63N,100.32E	0.000	0.000	Hap8(4)
DQ	5 (DQ01/DQ03/DQ05/DQ07/DQ08)	28.44N,98.89E	0.240	0.900	Hap3(1), Hap4(2), Hap23(1), Hap24(1)
FSX	5 (FSX01/FSX03/FSX05/FSX06/FSX07)	28.79N,98.66E	0.260	0.700	Hap14(1), Hap23(3), Hap45(1)
HS	8 (HS01- HS08)	32.07N,103.06E	0.120	0.786	Hap56(1), Hap58(1), Hap59(4), Hap60(1), Hap61(1)
MK	4 (MK01- MK04)	31.84N,101.76E	0.080	0.500	Hap65(1), Hap66(3)
MWQ	10 (MWQ01- MWQ10)	31.18N,97.09E	0.010	0.778	Hap30(5), Hap31(1), Hap32(1), Hap33(1), Hap34(1), Hap35(1)
MX	7(MX01- MX07)	31.89N,103.46E	0.200	0.952	Hap49(1), Hap52(1), Hap53(1), Hap54(1),

					Hap55(5), Hap57(1)
SAS	4 (SAS01/SAS03/SAS05/SAS07)	26.67N,91.35E	0.000	0.000	Hap46(4)
SH53	5 (SH53_1/SH53_4/SH53_5/SH53_7/SH53_9)	28.66N,84.02E	0.000	0.000	Hap68(5)
SH78	5 (SH78_1/SH78_10/SH78_2/SH78_7/SH78_8)	29.23N,82.04E	0.040	0.600	Hap70(2), Hap71(3)
SX	6 (SX01/SX03/SX05/SX07/SX09/SX10)	28.22N,90.98E	0.000	0.000	Hap69(6)
WC	7 (WC01/WC02/WC03/WC04/WC06/WC07/WC08)	31.52N,103.53E	0.180	0.810	Hap50(3), Hap51(2), Hap62(1), Hap67(1)
YJ	6 (YJ001- YJ006)	30.03N,101.02E	0.120	0.733	Hap11(2), Hap12(3), Hap13(1)
ZK	5 (ZK01/ZK03/ZK05/ZK06/ZK07)	29.62N,98.35E	0.160	0.400	Hap9(1), Hap25(4)

Table S8 The contribution of 19 climatic variables to the distribution of *Mirabilis himalaica* and explanations of each climatic variable.

Variable	Environmental variable	Percent contribution (%)
Bio6	Min Temperature of Coldest Month (℃)	26.1
Bio8	Mean Temperature of Wettest Quarter (℃)	17.5
Bio12	Annual Precipitation (mm)	9.3
Bio18	Precipitation of Warmest Quarter (mm)	7.6

Bio3	Isothermality (Bio2/Bio7) (×100)	6.5
Bio2	Mean Diurnal Range (Mean of monthly (max temp - min temp)) (℃)	5.9
Bio11	Mean Temperature of Coldest Quarter (℃)	5.4
Bio7	Temperature Annual Range (Bio5-Bio6) (℃)	5.3
Bio1	Annual Mean Temperature (℃)	3.5
Bio4	Temperature Seasonality (standard deviation ×100)	2.7
Bio15	Precipitation Seasonality (Coefficient of Variation)	1.7
Bio5	Max Temperature of Warmest Month (℃)	1.7
Bio9	Mean Temperature of Driest Quarter (℃)	1.6
Bio16	Precipitation of Wettest Quarter (mm)	1.5
Bio19	Precipitation of Coldest Quarter (mm)	1.5
Bio10	Mean Temperature of Warmest Quarter (℃)	1.1
Bio14	Precipitation of Driest Month (mm)	0.5
Bio17	Precipitation of Driest Quarter (mm)	0.3
Bio13	Precipitation of Wettest Month (mm)	0.1
